# Developmental and Immune Role of a Novel Multiple Cysteine Cluster TLR From *Eisenia andrei* Earthworms

**DOI:** 10.3389/fimmu.2019.01277

**Published:** 2019-06-18

**Authors:** Petra Prochazkova, Radka Roubalova, Frantisek Skanta, Jiri Dvorak, Natividad Isabel Navarro Pacheco, Miroslav Kolarik, Martin Bilej

**Affiliations:** ^1^Laboratory of Cellular and Molecular Immunology, Institute of Microbiology of the Czech Academy of Sciences, Prague, Czechia; ^2^Laboratory of Fungal Genetics and Metabolism, Institute of Microbiology of the Czech Academy of Sciences, Prague, Czechia

**Keywords:** earthworm, innate immunity, PRR, TLR, invertebrate, parasite, gregarine, development

## Abstract

Earthworms are not endowed with adaptive immunity and they are rely on the tools of innate immunity. Cells of the innate immune system utilize pattern recognition receptors, such as Toll-like receptors, to detect the pathogen-associated molecular patterns (PAMPs). The first earthworm TLR was isolated from *Eisenia andrei* earthworms (*Ea*TLR), which belongs to the single cysteine cluster TLR (sccTLR). Here, we identified a new multiple cysteine cluster TLR (mccTLR) in *E. andrei* earthworms. Phylogenetic DNA analysis revealed that it has no variability within one earthworm as well as in the population. By screening of the tissue expression profile, the TLR was expressed primarily in earthworm seminal vesicles and receptacles suggesting a connection to sperm cells. Seminal vesicles are often heavily infected by gregarine parasites. As a sign of immune response, a strong melanization reaction is visible around parasites. Stimulation experiments with profilin from related parasite *Toxoplasma gondii*, led to the upregulation of mcc*Ea*TLR in the earthworm seminal vesicles. Also, profilin activated prophenoloxidase cascade, the efficient mechanism of innate immunity. However, its involvement in the NF-κB signaling was not proven. Further, we provide evidence that the antibiotics metronidazole and griseofulvin destroyed the developing spermatocytes. The observed decrease in the mcc*Ea*TLR mRNA levels after the antibiotic treatment of parasites is caused by the decline of sperm cells numbers rather than by diminution of the parasites. Since earthworms with extensively reduced parasite load had a similar amount of mcc*Ea*TLR mRNA, presumably, earthworm sperm cells have a certain level of mcc*Ea*TLR expressed as a standard, which can be augmented by particular antigenic stimulation. Also, mcc*Ea*TLR was expressed mainly in the early stages of earthworm development and presumably is primarily involved in early embryonic development. Expression of mcc*Ea*TLR in seminal vesicles correlates with the expression of endothelial monocyte-activation polypeptide II. High-throughput sequencing of gregarine DNA from seminal vesicles of individual earthworms resulted in great diversity of the observed genotypes. Phylogenetically, all observed OTUs belong to the clade of earthworm gregarines suggesting host specificity. Overall, mcc*Ea*TLR is supposed to play a function role in early embryonic development and potentially it participates in immune response against parasites.

## Introduction

Invertebrates have developed a number of defense mechanisms that efficiently recognize and eliminate foreign materials, microbes, or parasites. They lack adaptive immunity based on the presence of antibodies, and lymphocytes and they rely primarily on innate immunity mechanisms that are often based on pattern recognition receptors (PRRs) (1).

In earthworms, three types of PRRs have been described so far—Toll-like receptor *Ea*TLR, coelomic cytolytic protein CCF, and lipopolysaccharide-binding protein *Ea*LBI/BPI (2–4). TLRs are conserved membrane pattern recognition receptors that detect microbes on the basis of pathogen-associated molecular patterns (PAMPs) (5). The first member of this family, named Toll, was originally identified as a molecule responsible for the embryonic dorsoventral development of the fruit fly *Drosophila melanogaster* (6), and later, its role in the protection against fungi and Gram-positive bacteria was discovered (7). Generally, TLRs are membrane glycoproteins consisting of three domains: the extracellular N-terminal domains with leucine-rich repeats (LRRs) responsible for the binding of antigens, the transmembrane domain, and the intracellular domain known as the Toll/IL-1 receptor (TIR) domain, required for the interaction and recruitment of various adaptor molecules to activate the downstream signaling pathway (8). Animal TLRs can be categorized into two major types based on the number of cysteine cluster on the C-terminal end of LRRs: single cysteine cluster TLR (sccTLR) and multiple cysteine cluster TLR (mccTLR) (9) The sccTLRs include all described vertebrate TLRs and a minority of insect TLRs. The mccTLRs comprise nearly all genes found in insects [e.g., Toll itself and other protostomes, e.g., *C. elegans* Tol-1 (10)]. Some authors use other terms for both structural classes, namely protostome- like type (equivalent of mccTLR) and deuterostome-like type (equivalent of sccTLR) (11).

The first earthworm TLR was isolated from an oligochaete annelid *Eisenia andrei* (*Ea*TLR) (3), which belongs to the single cysteine cluster (vertebrate) TLR type. This receptor has very large intraspecies variability, suggesting the presence of a large number of TLR genes within the *E. andrei* genome. Phylogenetic analysis revealed the great similarity of *Ea*TLR with a TLR from the polychaete annelid *Capitella teleta*, and with TLRs of mollusks and echinoderms. *Ea*TLR is expressed in all tissues of the earthworm body with the greatest constitutive expression in the digestive tract. Further, its expression in coelomocytes can be upregulated by the bacterial challenge (3).

Endothelial monocyte-activating polypeptide II (EMAPII) is a proinflammatory cytokine and chemoattractant for monocytes. In apoptotic cells, pottranslational processing of pro-EMAP/p43 to the mature EMAPII occurred coincidentally with apoptosis (12). It was shown that TLRs regulate EMAPII production upon microbial challenge in both mammals (13) and leech (14). Recently, an earthworm ortholog of the vertebrate complex p43/EMAPII was sequenced in our laboratory (GenBank: AEB92227).

Between defense molecules described in earthworms belong antimicrobial proteins with hemolytic activity—Fetidin and Lysenin (15–17). It was later confirmed that fetidin and lysenin represent two distinct molecules with similar properties encoded by two different genes (18). Changes in the composition of the microbial environment result in variations of fetidin and lysenins mRNA levels in the coelomocytes of *E. andrei* (19). Also, lysenin was described to bind sphingomyelin in the cell wall (20).

In the earthworm community, there is a large incidence of gregarine infection in seminal vesicles. Gregarines belong to the Apicomplexa group and represent extracellular parasites that inhabit the coeloms, the intestines and reproductive organs of marine, freshwater, and terrestrial invertebrates. Earthworms primarily host *Monocystis* sp. Von Stein 1848. The genus *Monocystis* is characterized by having a symmetric short or elongated body, ovoid gamonts, spores with typical fusiform shape and no mucron (21). Over 190 species belonging to the family Monocystidae have been described from invertebrates all over the world, 106 species are in the family Lumbricidae (21). Earthworms are infected by consuming a sporocyst with soil; released sporozoites in the digestive tract penetrate the intestinal wall and enter the dorsal blood vessel. From the vessel, they enter the seminal vesicles and feed on the developing spermatocytes in the wall of the seminal vesicle. In the lumen, they mature into trophozoites, which pair up in syzygy and develop into gamonts. After a couple of divisions, developed gametes fuse and form zygotes, which are surrounded by oocyst walls. Oocysts, full of sporocysts, are then released through the male genital pore to the soil (22).

Spermatogenesis in earthworms takes place in reproductive organs called seminal vesicles. Sperm morula change into the spermatocytes and spermatids, which are clustered around the cytophore. Each germ cell is connected to the cytophore via the cytoplasmatic bridge. Mature sperm cells are then released. Potentially, the spermatocyte is infected by a gregarine sporozoite eating the cytoplasm, and matures into the trophozoite with sperm filaments on the surface (23).

The prophenoloxidase-activating system is a sensitive non-self-recognizing cascade triggered by components of microbial cell walls such as lipopolysaccharides, peptidoglycans, and β-1,3-glucan (24, 25). Phenoloxidase is usually present in an inactive form, prophenoloxidase (proPO), in cells or body fluid of different invertebrate species. Conversion of proPO to its active state is achieved by proteolytic cleavage that depends on a cascade of serine proteinases, so-called the prophenoloxidase activating enzymes (ppA), and other factors. Then, PO catalyses both the *o*-hydroxylation of monophenols and the oxidation of diphenols to quinones, which non-enzymatically polymerize into melanin (26, 27). Melanin, as a final product of proPO cascade, has fungistatic, bacteriostatic, and antiviral properties and is involved in the innate immune response of certain invertebrates, especially arthropods. In earthworms, melanization reactions proceed like cellular defense reactions of the host through the formation of brown bodies around encapsulated invading pathogens (28). PO activity was revealed in *Eisenia fetida* by incubating its coelomic fluid with constituents of microorganisms and PO substrate L-DOPA (29).

The immune recognition of gregarine parasites was already suggested earlier when it was shown that *Lumbricus terrestris* coelomocytes are capable of recognizing *Monocystis in vitro* as non-self (30).

In the present article, we describe a new multiple cysteine cluster TLR in the oligochaete *E. andrei* (mcc*Ea*TLR), which is expressed mainly in reproductive organs and which could be connected to the gregarine infection in earthworm seminal vesicles as well as to participate in embryonic development.

## Materials and Methods

### Breeding of Earthworms, Isolation of Coelomocytes and Coelomic Fluid, Preparation of Protein Lysates

Adult *E. andrei* earthworms (*Oligochaeta*, Annelida) were maintained on moist paper towels without food for 2 days to empty their digestive systems. Various developmental stages were reached by the breeding of earthworms from cocoons in an artificial substrate containing sterile coconut fiber with 1% oatmeal flour. Coelomic fluid containing free coelomocytes was obtained through sonication of three earthworms in 2 ml of *Lumbricus* balanced salt solution (LBSS; modified isotonic PBS diluted with water 3:2 v/v; pH 7.3) in an ultrasonic bath (37 kHz, 1.5 min) with subsequent centrifugation (500 g, 10 min, 4°C). Coelomocytes were then washed in LBSS and used for RNA isolation. The integrity of isolated coelomocytes was checked microscopically before further procedures. The survival rate of isolated coelomocytes was always at least 93%. Tissues (epidermis, esophagus, pharynx, crop, gizzard, intestine, seminal vesicles, seminal receptacles) were obtained by dissection of adult earthworms on a frozen plate under a microscope with sterile equipment. Tissues were isolated from five individual adult animals, homogenized with lysing matrix D (FastPrep 24, MP Biomedicals) and used for RNA isolation.

For the protein lysates, coelomocytes, and seminal vesicle tissues were lysed in T-Per Tissue protein extraction reagent (Thermo Scientific) with proteinase inhibitor Arrest (Thermo Scientific) in combination with bead-beating (lysing matrix D, MPG). After 30 min incubation on ice, samples were centrifuged for 20 min (14,000 rpm) and supernatants were used for PO activity assessment.

### Isolation of Genomic DNA

Genomic DNA was isolated from a body segment or from seminal vesicles using the MasterPure complete DNA & RNA purification kit (Epicentre) and FastPrep 24 homogenizer with Lysing Matrix Y (MP Biomedicals).

### Sequence and Phylogenetic Analyses

A partial *Lumbricus rubellus* sequence found in the LumbriBASE database (http://xyala.cap.ed.ac.uk/Lumbribase/lumbribase_php/lumbribase.shtml, Elsworth^1^) was used to design degenerated primers for *E. andrei* p-TLR amplification. The mcc*Ea*TLR sequence was obtained using the 3′- and 5′-RACE System (Life Technologies). The remaining sequence containing the signal sequence was obtained by inverse PCR from gDNA. Genomic DNA was firstly restricted by EcoRI with subsequent self-ligation. The obtained sequences were then restricted by BglII and the linearized DNA was amplified with specific inverse primers. The resulting products were cloned into pCR2.1-TOPO, sequenced, and submitted to the database (NCBI: LT219466). The domain organization was designed by the SMART program.

Genomic DNA and RNA from 20 individual earthworms were isolated. The obtained gDNA and cDNA were used in PCR using the P5*Ea*TLR/P*Ea*TLR5 primer pair (**Table 2**) to assess the intraspecies variability and to identify the presence of the introns. The primers utilized were designed according to the obtained mcc*Ea*TLR DNA sequence. All amplified PCR products formed overlapping contigs. Genomic DNAs and the resulting cDNAs were amplified by different primer sets covering the whole molecule, cloned into the pCR2.1-TOPO vector, and sequenced. Also, four different clones from each earthworm were sequenced and analyzed.

For phylogenetic analyses, the TIR domain of mcc*Ea*TLR and the TIR domains of TLRs deposited in the GenBank database were used (for accession numbers see **Figure 2**). The sequence selection was performed using tBlastX similarity search of mcc*Ea*TLR and *Ea*TLR sequences and the entries representing the complete taxonomic diversity of Animalia were used to assess the phylogenetic placement of our data. The alignments were obtained using MAFFT 6 (http://mafft.cbrc.jp/alignment/software/) (31). Only the more conservative TIR domains, which can be aligned unambiguously, were used in the analysis and the alignment was cured using Gblocks version 0.91b (32). The final dataset had 20 sequences and 297 amino acid residues. Maximum likelihood (ML) analyses were performed in PhyML 3.0 (33) using the WAG substitution model, and bootstrap support was obtained using 1,000 replicates. Evolutionary models were determined using datasets and MEGA 5.05 (34). The tree was rooted with *Ciona intestinalis*.

### RNA Isolation, cDNA Synthesis, qPCR, Preparation of Plasmids

Total RNA was isolated from coelomocytes, from various tissues or from whole body tissues of the individual earthworms, using TRIZOL reagent (Life Technologies) according to the manufacturer's protocol. One microgram of DNAse I treated total RNA was reverse-transcribed using the Oligo(dT)12–18 primer and Superscript IV Reverse Transcriptase (Life Technologies) and subsequently used in a PCR reaction. Non-RT controls were run in parallel to prove the elimination of gDNA contamination.

Quantitative PCR (CFX96 Touch™, Bio-Rad) was performed to determine changes in the mRNA levels of mcc*Ea*TLR *Ea*TLR, EMAP, LBP/BPI, Fet/Lys, NF-κB, and MyD88 (primers are shown in **Table 2**) (3, 4, 35). The cycling parameters were as follows: 4 min at 94°C, 35 cycles of 10 s at 94°C, 25 s at 60°C, and 35 s at 72°C, and a final extension for 7 min at 72°C. Changes in gene expression were calculated according to the $2^{-\Delta \Delta {\text{C}}_{\text{T}}}$ (Livak) method. Two reference genes (RPL13, RPL17) were selected as internal controls for the normalization of the expression of the other genes. The fold change in the mRNA level was related to the change in the settled controls. The results were expressed as the mean (±SD) of the values obtained in three independent experiments. Evidence of significant changes was evaluated using the one-sample *t*-test or two-way ANOVA with Bonferroni posttest in the GraphPad Prism software. The clustergram was evaluated using Bio-Rad CFX manager software.

Similarly, the absolute copy numbers of mcc*Ea*TLR and *Ea*TLR in samples were assessed by qPCR using reference plasmids. Partial sequences of the TIR domain of both molecules (PCR products of primer sets for qPCR, **Table 2**) were cloned into a pCR2.1 vector using a TA cloning kit (Life Technologies). Reference plasmids were propagated in competent *E. coli* NEB10-beta cells (New England Biolabs). Plasmid DNA was isolated with PureLink Quick Plasmid Miniprep Kit (Life Technologies), restricted with BamH1 to obtain the linear form, purified, and used in the concentration of 10^9^-10^1^.

### Treatment of Earthworms

Previously, two antibiotics, metronidazole, and griseofulvin, were considered as highly effective against arthropod gregarines (36). Earthworms (five worms in each group) were cured of parasitic infection with metronidazole (Sigma-Aldrich) or with griseofulvin (Sigma-Aldrich) at doses of 0.4–50 mg/worm by keeping them in an environment containing antibiotics for 15 days, with a new dose every 2 days. From the survival experiments, two doses were chosen for each antibiotic. From each earthworm, the entire lobes of the seminal vesicles were dissected, and either homogenized with lysing matrix D in RPMI medium (FastPrep 24, MP Biological) for gDNA/RNA isolation, or weighed, homogenized in Ringer solution (1 mg/10 ul), twice sonicated, and used for counting of sporocysts. In control non-treated and treated earthworms, the number of sporocysts, gregarine DNA content, and mRNA levels of both mcc*Ea*TLR and *Ea*TLR were assessed. The sporocyst concentration was determined with a Bürker counting chamber under a phase contrast microscope with 40 ×10 magnitude. Sporocyst counts were taken twice for each sample and averaged. To follow the gregarine content in seminal vesicles, specific primers amplifying different gregarine species (but not earthworm DNA) were designed, and together with an apicomplexan-specific reverse primer Api1R were used to amplify the 18S of the gregarines present (**Table 2**) (37). The specificity of the primers was proven by sequencing. Also, the gregarine specific primers were used to determine the gregarine abundance in various earthworm tissues. mRNA levels of both TLRs were determined by qPCR as described previously.

### “GF-Like” Earthworms

To prepare earthworms without parasite infection, which would be used as a negative control, we picked up 30 cocoons, which were cleaned with an antibiotic mixture (antibiotic-antimycotic solution 10x diluted, Sigma) and earthworms were bred to adulthood under semi-sterile conditions in the sterile artificial substrate. The gregarine content and mRNA levels of mcc*Ea*TLRs were then assessed.

### Stimulation of Earthworm Seminal Vesicles Tissue and Coelomocytes

Seminal vesicles from earthworms (six for each group) were dissected and put in RPMI medium containing L-glutamine, 5% FBS, 10 mM Hepes, 2 mM sodium pyruvate, and 1x antibiotic antimycotic solution (Sigma). Dissected tissues and isolated coelomocytes were then incubated with representative TLR ligands (Table 1) for 6 h. Isolated RNA was then used for the cDNA transcription and mRNA levels of assorted defense and signaling molecules were assessed.

**Table 1:** The TLR ligands used for the stimulation of coelomocytes and seminal vesicles tissue.

**Ligand**	**Working concentration**
*Toxoplasma gondii* inflammatory profilin recombinant protein (LifeSpan BioSciences)	100 ng/well
Poly I:C, synthetic analog of double stranded RNA (Invivogen)	10 ug/well
Lipoteichoic acid from *S. aureus* (Invivogen)	10 ug/well
Zymosan A (Sigma)	10 ug/well
Flagellin from *S. typhimurium* (Invivogen)	100 ng/well
LPS from *E. coli* O55:B5 (Sigma)	10 ug/well
ODN2006, synthetic oligonucleotides containing unmethylated CpG dinucleotides	100 nM/well

### Induction of Prophenoloxidase Activating System

Briefly, 10 μl of the sterile coelomic fluid, cell or seminal vesicles lysate (with or without 1 mM serine proteinase inhibitor coctail Recom ProteaseArrest (G-Biosciences), 80 μl of buffer (100 mM Tris, 50 mM CaCl_2_, pH 8) containing 10 mM L-DOPA (L-β-3,4-dihydroxyphenylalanine; Fluka) and 10 μl of either LSP (100 ug/ml; *E. coli* 055:B5 S strain, Sigma), inflammatory profilin recombinant protein (4 ug/ml, LifeSpan BioSciences), 0.05% cetylpyridiumchlorid as an non-specific activator or none activator was incubated at room temperature up to 7 h. The oxidation of L-DOPA to dopachrome was measured every hour at 490 nm. The absorbance of samples without CF or lysate was subtracted and then evaluated as the difference between the A_490_ values with or without proteinase inhibitor.

### Hematoxylin/Eosin Staining

Dissected seminal vesicles of tested earthworms were maintained in a drop of water on a microscopic slide (30 μl) which was then disrupted on the surface of the slide. The smear was allowed to air dry for 5 min. The slides were fixed in 96% ethanol for 15 min and stored in 70% ethanol. Staining with hematoxylin/eosin was performed according to the protocol (38).

### Toluidine Blue Staining

Fresh smears of dissected seminal vesicles were immediately stained in a fresh solution of toluidine blue (final concentration 0.1%) for 20 min and analyzed using a Carl Zeiss microscope.

### Confocal Microscopy

The specificity of the antibody binding was checked by western blot analysis of seminal vesicles protein lysate (Figure S1 in Supplementary Material). Seminal vesicles were gently disrupted directly onto a microscope slide and let air dried for 5 min. Tissue smear was fixed in cold methanol for 15 min at −20°C. Samples were treated by blocking buffer (1 x PBS, 5% normal donkey serum, 0.3% Triton^TM^ X-100) for 1 h at RT. Samples were incubated with primary monoclonal antibody NF-κB p65 (#8242, Cell Signaling Technology) diluted 1:100 (1xPBS, 1% BSA, 0.3% Triton^TM^ X-100) overnight at 4°C. Next, samples were incubated with secondary Alexa Fluor® 488 Donkey anti-rabbit IgG polyclonal antibody (Biolegend, cat. n.: 406416) diluted 1:200 for 2 h in dark followed by DAPI (1.5 μg/ml) staining for nucleic acid detection. Specimens were examined immediately using appropriate excitation wavelength under a confocal laser scanning microscope. Images were analyzed by Olympus Fluoview.

### High Throughput Sequencing

Total extracted gDNA from earthworm seminal vesicles was used for high throughput sequencing (Miseq platform, Illumina) of the 18S rRNA gene of gregarines in 12 individual earthworms. Two sets of specific primers with barcodes were used in PCR (Table S1 in Supplementary Material). PCR amplifications (KAPA 2G Robust Hot Start DNA Polymerase, Kapa Biosystems) were carried out with 27 cycles. The PCR products were purified and normalized with the SequalPrep™ Normalization Plate Kit (ThermoFisher Scientific). Triplicates of the amplicons were pooled and ligated with sequencing adapters (TruSeq DNA PCR-free LT Sample Preparation Kit, Illumina), pooled in equimolar concentrations, and sequenced. The library was validated by a KAPA Library Quantification Kit (Illumina). The amplicons were sequenced on an Illumina MiSeq using a Miseq Reagent Kit v3 (Illumina).

Data collected from sequencing runs were processed using the Qiime pipeline applying standard procedures such as through quality control and data filtering, clustering analysis, and diversity determination (39).

### Sequence Data Processing and Phylogenic Analysis of Gregarine SSU rDNA Analysis

Two different regions of gregarine SSU (regions of 200 and 477 bp) were generated by PCR with the use of specific primers, which were designed to amplify most terrestrial gregarines (available in NCBI databases), but not earthworm DNA (Table 2).

**Table 2:** Primers used in sequence analysis and qPCR.

**Name**	**Direction**	**sequence**	**Product size (bp)**
**Primers for sequence analysis**			
mcc5*Ea*TLR	Forward	5′-TTAGCAACGTATCAGCAAATCT-3′	
mcc*Ea*TLR5	Reverse	5′-GTTAGGTTCAGTCTCCAAAGGTAT-3′	1978
**Primers for qPCR**			
mcc*Ea*TLR	Forward	5′-CATCCCAGAATACAATCCAAACAG-3′	233
	Reverse	5′-ATACTGACGGTCCGGAAAGAAAAT-3′	
*Ea*TLR	Forward	5′-GAGATATCGCTGAAAACATCCTG-3′	215
	Reverse	5′-CTGCATCTGAATCTGGAGTCTTG-3′	
RPL17	Forward	5′-CATCACACCCTACATGAGCA-3′	179
	Reverse	5′-TAACGGAAGAAGGGGTTAGC-3′	
RPL13	Forward	5′-CACAATTGGAATTGCTGTCG-3′	144
	Reverse	5′-GTGGCATCACCCTTGTTAGG-3′	
EMAP	Forward	5′-CATCCCGATGCGGACAGTCTGTA-3′	244
	Reverse	5′-TCCCCAATGGCAGCACCAATT-3′	
LBP/BPI	Forward	5′-GACCAATCTGCCTGCGAGTTC-3′	227
	Reverse	5′-CAGGGCGTCCATTATCTACATCAC-3′	
Fet/Lys	Forward	5′-TGGCCAGCTGCAACTCTT-3′	176
	Reverse	5′-CCAGCGCTGTTTCGGATTAT-3′	
NF-κB	Forward	5′-CAAATGCGAAGGACGATCCG-3′	112
	Reverse	5′-CACTATTGCACCCGGACCAT-3′	
Myd88	Forward	5′-CAGGTGCCAAGGAGAAGAAG-3′	167
	Reverse	5′-TAGTTGGGAGATCGGGAATG-3′	
**Primers for gregarine 18S rRNA**			
SSU1f	Forward	5′-CCATGCATGTCTAAGTATAAGTT-3′	200
SSU1r	Reverse	5′-TGCAAGCATAGGTTGGTTCT-3′	
SSU2f	Forward	5′-AGTTGTCAATCAAATGACTCTTTC-3′	477
Api1r	Reverse	5′-TAATCTATCCCCATCACGATGC-3′	

*X represents a barcode base, linkers are in italic*.

The amplicon sequencing data were processed with SEED v2.1 (40). Pair-end reads were joined using fastq-join (41). Chimeric sequences were detected using algorithm UCHIME, deleted and clustered using UPARSE at a 97% similarity level, both of which were conducted with USEARCH 8.1.1861 (42). The most abundant sequences were chosen as one representative strain per cluster. The dataset supporting the conclusions of this article (raw demultiplexed sequencing data with sample annotations), is available in the Sequence Read Archive of EMBL (PRJNA494629) (43). Singletons were excluded from all analyses. The clustered sequences obtained from longer SSU regions (SSU2/Api region, 477 bp) were used for phylogenetic analysis. Sequences were compared with data from the NCBI GenBank using a BlastN similarity search (see **Figure 10** for sequence accession numbers) including those published by Leander et al. (44). A matrix containing SSU sequences were aligned in MAFFT 6 using the G-INS-i strategy (45). The final alignment contained 49 sequences and 543 characters, of which 222 were conserved, 251 were variable, and 159 parsimony-informative. The maximum likelihood (ML) phylogenetic analyses were performed in PHYML (33) using default settings and 500 bootstrap replicates with the K2 + G model determined using MEGA 6.06 (34). The tree was rooted with *Cryptosporidium muris*, an Apicomplexan species related to gregarines (46).

### Diversity of Gregarine SSU rDNA

The pipeline SEED v2.1 (40) was used for the calculation of α-diversity (Shannon-Wiener index), species richness, evenness, rarefaction plots, and Chao 1 indexes using 6,000 randomly selected sequences from each sample.

## Results

### Sequence Characterization

**Figure 1:**
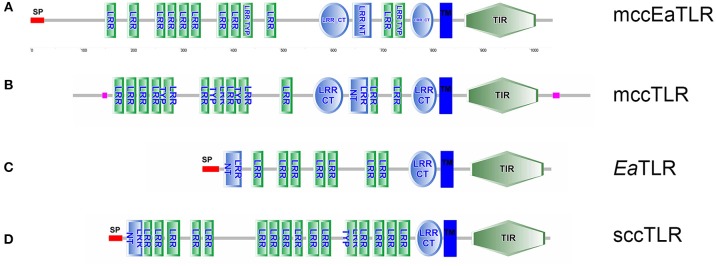
Protein domain structure of two types of *Ea*TLR. **(A)** Protein domain structure of mcc*Ea*TLR (NCBI: CZQ50134), **(B)** typical mccTLR (*Drosophila melanogaster*; NCBI: AAQ64937), **(C)** *Ea*TLR (NCBI: JX898685), and **(D)** typical sccTLR (*Branchiostoma lanceolatum*; NCBI: AXP19712). SP, signal peptide; LRR, leucine-rich repeats; LRR Typ, LRR Typical Subfamily; LRR-NT, N-terminal LRR domain; LRR CT, C-terminal LRR Domain; TIR, Toll-Interleukin-1 Receptor Domain; TM, Trans-membrane Region. Different domains were detected by SMART.

By screening of the LumbriBASE *EST* database (Elsworth^1^) we found a partial sequence revealing homology with the TIR domain of the *Drosophila* Toll molecule. Degenerated primers designed based on this sequence were used to obtain an *E. andrei* cDNA fragment, which was cloned and sequenced. In order to assemble the full-length cDNA, RACE amplifications of 5′and 3′ ends were performed. The remaining portion of the 5′-end, impossible to obtain by the RACE system, was achieved by inverse PCR. Consequently, cDNA of 3,858 bp containing a 439 bp 5′-UTR, 278 bp 3′-UTR with polyadenylation signal sequence (ATTAAA) at the 3,475 bp position, and a poly(A)-tail was obtained. An open reading frame (ORF) of 3,141 bp encoding a putative protein of 1,046 amino acids was observed (the whole sequence can be found in the NCBI database: LT219466). The mcc*Ea*TLR gene has no introns as proved by PCR and following the sequencing of 20 individual earthworm gDNA fragments. The pI was predicted as 5.99, and the molecular weight was calculated as 118.6 kDa. The mcc*Ea*TLR protein exerts 18 predicted N-glycosylation sites. SMART™ protein domain analysis revealed that the deduced protein harbors an intracellular TIR domain (143 aa), a transmembrane domain (22 aa), 12 internal extracellular LRRs (23–24 aa), 2 LRR-C-terminal domains (46 and 60 aa), and 1 LRR-N-terminal domain between them (38 aa) (Figure 1). In contrast to the “vertebrate-like” type with cysteine-rich N- and C-terminal LRR motifs (LRR-NT/CT) capping both ends of the internal LRR solenoid, the mcc*Ea*TLR molecule shares typical features of a “protostome-like” type TLR containing an ectodomain where an internal LRR-CT and LRR-NT pair divides the LRR solenoid (Figure 1). In addition, an N-terminal signal peptide of 26 amino acid residues was predicted.

### Phylogenetic Analyses and Variability of mccEaTLR

**Figure 2:**
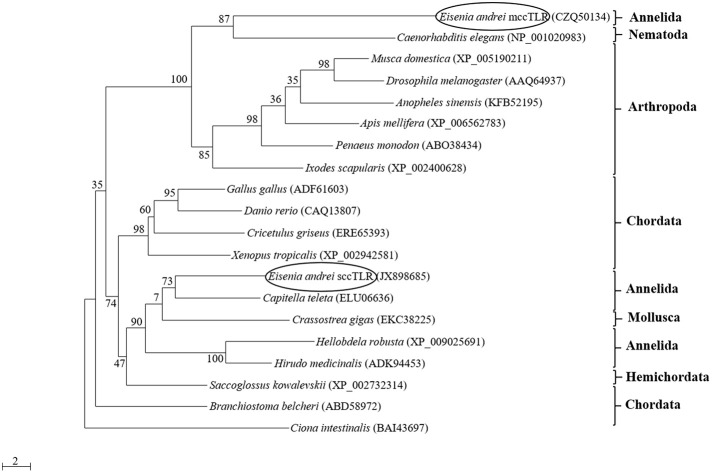
Maximum-likelihood phylogenetic analysis of new multiple cysteine cluster (mcc*Ea*TLR) and single cysteine cluster TLRs (*Ea*TLR) amino acid sequences. Sequence alignments were obtained using MAFFT 6, and maximum likelihood analyses was performed in PhyML 3.0 using the WAG substitution model. Bootstrap values (expressed as percentages of 300 replications) are shown at the branching points. Evolutionary model was determined using MEGA 5.05. The tree is rooted with *Ciona intestinalis*. Accession numbers of the sequences are indicated in parentheses.

The phylogenetic analysis of the amino acid sequences of TIR domains of 20 TLRs covering various animal groups (both multiple cysteine cluster and single cysteine cluster TLR from invertebrates and vertebrates) disclosed the high level of homology of mcc*Ea*TLR with the mccTLR of arthropods, and it forms one common clade with the TLR of *Caenorhabditis elegans* (Figure 2). The analysis of the intraspecies variability of the whole mcc*Ea*TLR molecule in 20 individuals resulted in no sequence variability observed within one earthworm as well as in the population.

### Tissue and Development Expression Profile of Both Earthworm TLR

**Figure 3:**
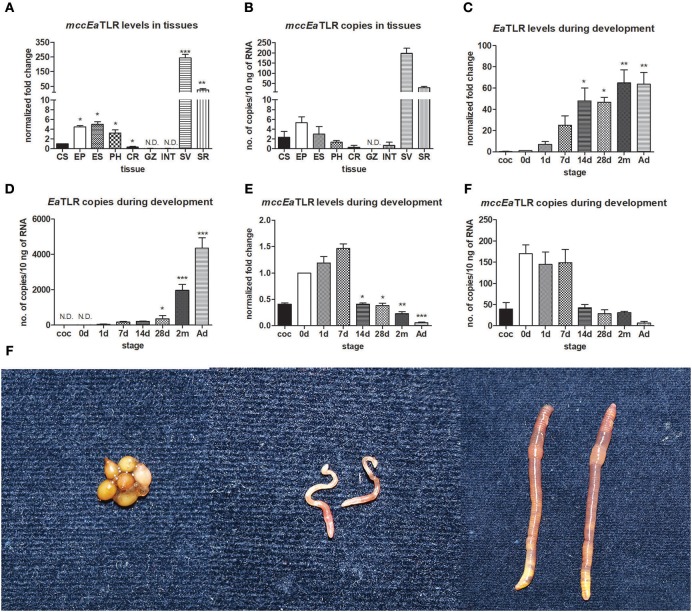
mRNA levels and absolute copy numbers of mcc*Ea*TLR and EaTLR in tissues and during development. **(A)** mcc*Ea*TLR mRNA levels in tissues, **(B)** mcc*Ea*TLR copies in tissues, **(C)** *Ea*TLR (scc*Ea*TLR) mRNA levels during development, **(D)** *Ea*TLR copies during development, **(E)** mcc*Ea*TLR mRNA levels during development, **(F)** mcc*Ea*TLR copies during development, **(G)** pictures of different *E. andrei* developmental stages—cocoons, 7 d old juveniles, adults. The values represent fold changes relative to levels in cells or to the just hatched earthworms (interval 0 d) (here settled as a value 1). Values are the means of two experiments (±SD) performed in triplicate. The significance was evaluated by one-sample *t*-test in the GraphPad Prism software (^*^P < 0.05; ^**^P < 0.01; ^***^P < 0.001). CS, coelomocytes; EP, epidermis; ES, esophagus; PH, pharynx; CR, crop; GZ, gizzard; INT, intestine; SV, seminal vesicles; SR, seminal receptacles; coc, cocoons; 0 d, just hatched earthworms; 1 d-2 m, the age of earthworms in days/month; Ad, adults.

To investigate the tissue expression profile of mcc*Ea*TLR, qPCR was performed on various tissues and cells. As shown in Figure 3, mcc*Ea*TLR is expressed mainly in seminal vesicles and seminal receptacles. The comparison of absolute numbers of mcc*Ea*TLR copies revealed its very low levels. Although the mcc*Ea*TLR mRNA was also found in anterior parts of the digestive tract, it is most likely due to their close position to the lobes of the seminal vesicles (Figures 3A,B). To estimate the role of TLRs during earthworm development, the mRNA levels of both TLRs were assessed in the whole body of differently aged specimens. The *Ea*TLR was expressed mainly in older stages, from around 14 days of age (Figures 3C,D), while mcc*Ea*TLR was expressed mainly in the early stages of earthworm development (Figures 3E,F). Similar to its expression in tissues, its absolute levels are much less than those of *Ea*TLR.

### Gregarines in Seminal Vesicles of Earthworms

**Figure 4:**
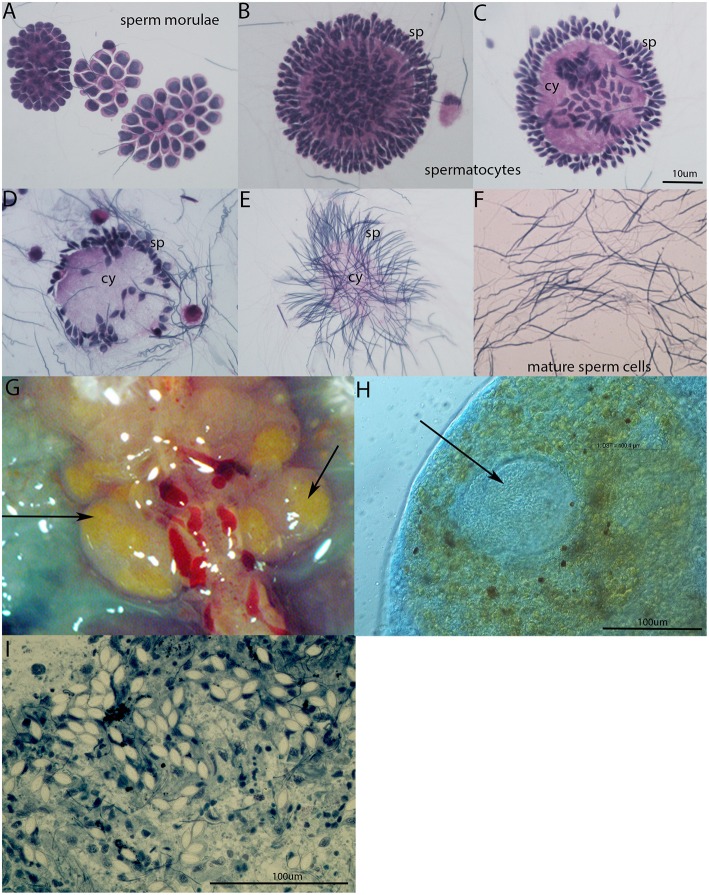
Spermatogenesis in *E. andrei* earthworms and gregarine infection in seminal vesicles. **(A)** Sperm morulae, **(B,C)** developing spermatocytes, **(D,E)** spermatids clustered around the cytophore, **(F)** mature sperm cells. Stained by Hematoxylin/Eosin. **(G)** Melanization reaction around the parasites in SV is visible as yellow area without any staining, **(H)** oocyst (indicated by an arrow) containing a large number of sporocysts surrounded by tissue with a melanization reaction clearly visible as brown spots, **(I)** individual sporocysts within the SV tissue, stained by Methylene Blue.

In the seminal vesicles of earthworms, different developing stages of spermatogenesis can be found (Figures 4A–F). Spermatogenic stages develop around an anucleate cytophore from which they separate as mature spermatozoa. During sperm maturation, some sperm morulae are infected by gregarine parasites. In our laboratory earthworm population, all tested specimens were positive for gregarine infection. The strong melanization reaction is visible around parasites in seminal vesicle tissue (Figure 4G). It is clearly visible as a yellow area in SV without any staining. The occurrence of the melanization reaction is a result of a prophenoloxidase cascade, an important defense mechanism of most invertebrates (24, 29). The further examination of the yellow parts of SV revealed the presence of the most distinguishable parasitic stage oocyst containing an abundance of sporocysts with a typical lemon-shape (Figures 4H–I).

### Treatment of Gregarine Infection With Antibiotics

**Figure 5:**
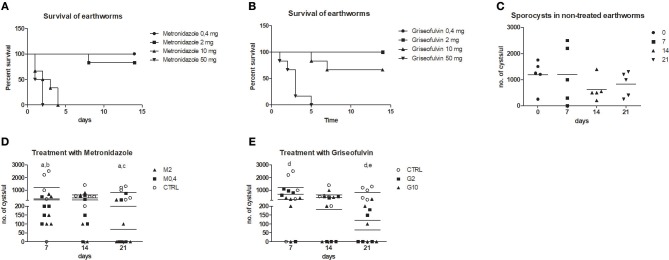
Treatment of parasitic infection by metronidazole and griseofulvin. Earthworms were treated with two types of antibiotics in a concentration range (0.4–50 mg/worm: M0.4,−50, G0.4-50) during 15 days and earthworm survival as well as the number of sporocysts were evaluated. **(A)** survival after the metronidazole treatment, **(B)** survival after the griseofulvin treatment, all control worms without any treatment survived (not shown in the graphs) **(C)** number of sporocysts in non-treated earthworms, **(D)** number of sporocysts in seminal vesicles after treatment with 0.4 and 2 mg/worm of metronidazole, **(E)** number of sporocysts in seminal vesicles after treatment with 2 and 10 mg/worm of griseofulvin. The significance was evaluated by one-sample *t*-test in the GraphPad Prism software. Different letters indicate significant differences between treatments and controls: a- M0.4 (*P* < 0.05); b- M2 (*P* < 0.05); c-M2 (*P* < 0.001); d- G2 (*P* < 0.01), e- G10 (*P* < 0.01).

**Figure 6:**
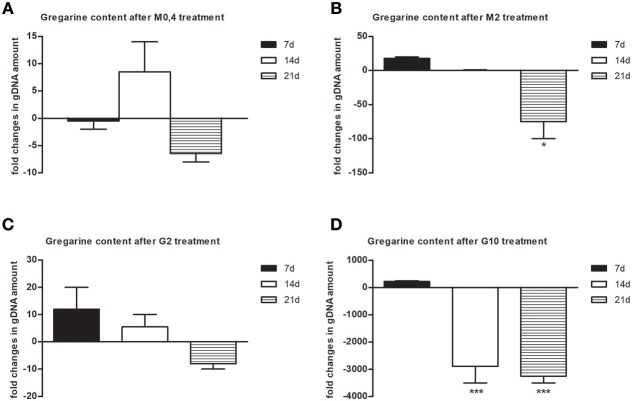
Gregarine DNA content in seminal vesicles after treatment with metronidazole and griseofulvin. Gregarine gDNA amount in seminal vesicles after treatment with 0.4 mg/ml **(A)** or 2 mg/ml metronidazole **(B)**, or with 2 mg/ml **(C)** or 10 mg/ml griseofulvin **(D)**, expressed as a fold change related to gDNA levels in control non-treated earthworms during 21 days, was assessed by qPCR. The significance was evaluated by one-sample *t*-test in the GraphPad Prism software (^*^P < 0.05; ^***^P < 0.001).

**Figure 7:**
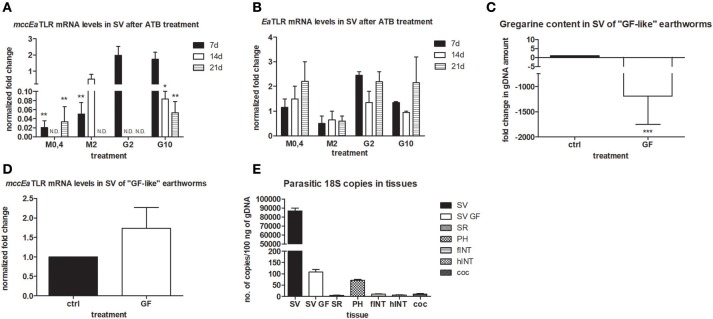
mRNA levels of both TLRs in seminal vesicles after antibiotic treatment, mRNA level of mcc*Ea*TLR in and gregarine content in seminal vesicles of “GF-like” earthworms and parasitic load of different tissues. **(A)** mRNA level of mcc*Ea*TLR, and **(B)** -*Ea*TLR in seminal vesicles after antibiotic treatment for 21 days was assessed by qPCR and related to mRNA levels in non-treated earthworms. The significance was evaluated by two-way ANOVA with Bonferroni posttest in the GraphPad Prism software (^*^P < 0.05; ^**^P < 0.01). **(C)** gregarine gDNA content in seminal vesicles of “GF-like” earthworms related to content in control earthworms. The significance was evaluated by one-sample *t*-test in the GraphPad Prism software (^***^P < 0.001). **(D)** mRNA levels of mcc*Ea*TLR in seminal vesicles of “GF-like” earthworms related to mRNA levels in control earthworms, **(E)** number of gregarine 18S copies in various tissues per 100 ng of isolated gDNA. Values are the means of two experiments (±SD) performed in triplicate. SV, seminal vesicles; SV GF, seminal vesicles of “GF-like” earthworms; SR, seminal receptacles; PH, pharynx; fINT, fore intestine; hINT, hind intestine; coc, cocoons.

To follow the connection of the gregarine presence in earthworm seminal vesicles (SV) with mcc*Ea*TLR, we treated worms with two types of antibiotics previously described as efficient substrates for gregarine infection in grasshoppers. From the survival experiments, we chose two concentrations for each antibiotic and we treated worms for 3 weeks (Figures 5A,B). All earthworms without any treatment survived throughout the whole experiment. The number of sporocysts in the control earthworms didn't vary significantly during the experiment, and their average concentration was about 700–1,100 sporocysts/ul of homogenate, corresponding to 100 μg of SV tissue (Figure 5C). In the treated groups, there was a moderate decrease in the concentration of cysts, mainly after the use of greater doses of antibiotics after 3 weeks of treatment (Figures 5D,E). To follow the content of parasites by qPCR, we designed primers amplifying only parasite DNA covering different gregarine species (Table 2). After 3 weeks of treatment, a decrease in the amount of parasite DNA was observed in all experimental groups, primarily after treatment with greater doses of antibiotics (Metronidazole 2 mg/ml and Griseofulvin 10 mg/ml; Figure 6). The measurement of the mRNA levels of both TLRs in the seminal vesicles of treated earthworms revealed that *Ea*TLR levels were not affected by the antibiotic treatment, but mcc*Ea*TLR was strongly downregulated, in some cases even undetectable (Figures 7A,B). Unfortunately, microscopic analysis of seminal vesicles from treated earthworms revealed a robust decrease in sperm cells, suggesting the doses of antibiotics utilized destroyed the developing spermatocytes (Figure S2 in Supplementary Material). Since the *Ea*TLR levels were not affected, it is presumed to be expressed on other cell types of seminal vesicles rather than on sperm cells, (e.g., epithelial cells).

Unexpectedly, “germ-free-like” bred earthworms, which were supposed to be used as a negative control, contained gregarine DNA in their seminal vesicles, although in much lesser amounts than in traditionally bred earthworms (proved by sequencing, Figure 7C). Moreover, the mRNA levels of mcc*Ea*TLR were similar in both traditionally bred and “germ-free-like” worms, indicating no correlation between the amounts of gregarine parasites and mcc*Ea*TLR expression (Figure 7D). While “germ-free-like” and traditionally bred earthworms differed considerably in their parasite load, their mcc*Ea*TLR levels in seminal vesicles were similar.

Further, the assessment of the amount of parasite DNA in various tissues also revealed its presence in some digestive tract tissues and a fractional amount in cocoons. Most likely, these gregarines are transferred during the cocoon formation (Figure 7E).

### Antigenic Stimulation of Seminal Vesicles Tissue and Coelomocytes

**Figure 8:**
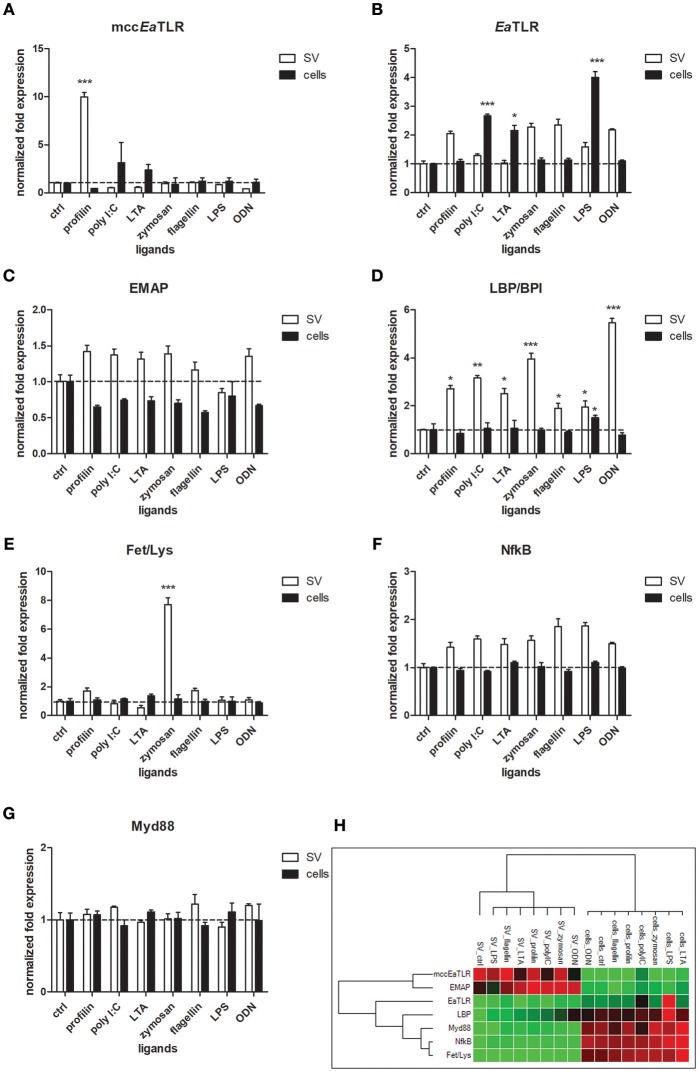
mRNA levels of selected immune and signaling molecules in seminal vesicles and coelomocytes after the stimulation with representative TLR ligands. Coelomocytes and seminal vesicles from earthworms were incubated for 6 h with various TLR ligands: profilin, poly I:C, lipoteichoic acid (LTA), zymosan, flagellin, LPS, ODN. The mRNA levels of **(A)** mcc*Ea*TLR, **(B)** *Ea*TLR, **(C)** EMAP, **(D)** LBP/BPI, **(E)** Fet/Lys, **(F)** NF-κB, and **(G)** Myd88 molecules were assessed. The values represent fold changes relative to non-treated controls (here settled as a value 1, dashed line). Values are the means of three experiments (±SD) performed in triplicate. The significance was evaluated by two-way ANOVA with Bonferroni posttest in the GraphPad Prism software (^*^P < 0.05; ^**^P < 0.01; ^***^P < 0.001). **(H)** Clustergram of differential gene expression in coelomocytes and seminal vesicles. Color ranges from green to red, through black, according to the magnitude of relative gene expression. Target are clustered according to their similarity in the expression pattern.

To reveal the involvement of mcc*Ea*TLR in the immune response, seminal vesicles, and coelomocytes were co-cultivated with various TLR antigens for 6 h and the changes in mRNA levels of assorted defense and signaling molecules were assessed. Seminal vesicles tissue exerted increased levels of mcc*Ea*TLR after the stimulation with profilin antigen from a related parasite *Toxoplasma gondii* (Figure 8A), which was identified as an antigen for mouse TLR11 and 12 (47). Presumably, earthworm sperm cells have a certain level of mcc*Ea*TLR expressed as a standard, which can be augmented transiently in the case of demand. Firstly described single cysteine cluster *Ea*TLR was augmented only in coelomocytes after the stimulation with poly I:C, lipoteichoic acid and LPS (Figure 8B). Interestingly, potential earthworm cytokine EMAP was found to be expressed in greater quantities seminal vesicles tissue than in coelomocytes. However, the expression was not affected by the antigenic stimulation (Figures 8C,H). Another pattern recognition molecule LBP/BPI was upregulated mainly in SV after the treatments with all used antigens. Only a slight increase was detected in coelomocytes after the activation with LPS (Figure 8D). Antimicrobial molecules Fet/Lys were augmented only in SV after the stimulation with zymosan (Figure 8E). Fet/Lys molecules were described to bind sphingomyelin in the cellular membrane (20). Zymosan, a component of the yeast cell wall, contain related lipid phytosphigosine (48), and most likely it can evoke the increase of Fet/Lys mRNA. mRNA levels of two signaling molecules NF-κB and MyD88 were not affected by any stimulants (Figures 8F,G). Clustrogram of all tested molecules shows separate relative expression of both tested niches—seminal vesicles and coelomocytes (Figure 8H). Mcc*Ea*TLR and EMAP were expressed mainly in SV tissue, whereas other tested molecules had higher abundance in coelomocytes.

### Phenoloxidase Activity in Coelomic Fluid and Seminal Vesicles and Cell Lysates

**Figure 9:**
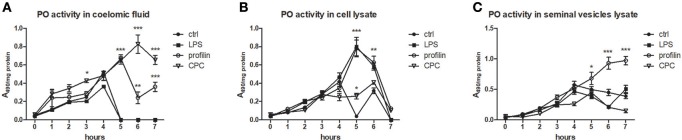
Activation of proPO system in earthworm celomic fluid, cells and seminal vesicles. ProPO activation of **(A)** coelomic fluid, **(B)** coelomocyte lysate supernatant, and **(C)** seminal vesicles lysate supernatant was assessed after the preincubation *in vitro* with LPS, profilin and CPC during 7 h. The significance was evaluated by two-way ANOVA with Bonferroni posttest in the GraphPad Prism software (^*^P < 0.05; ^**^P < 0.01; ^***^P < 0.001).

To analyze the ability of some activators to trigger the activation of prophenoloxidase cascade, we measured PO activity in coelomic fluid, cell lysate and seminal vesicles lysate during 7 h after the cocultivation with all types of samples with LPS, profiling or non-specific activator CPC. All tested type of control samples exerted the highest PO activity after the 4 h. We detected the highest increase of PO activity after the treatment with non-specific activator CPC after the 6 h and after the treatment with profilin after the 5 h (Figure 9A). In cell lysate, both LPS and profilin were able to strongly activate the proPO cascade after the 5 h (Figure 9B). In seminal vesicles lysate, only treatment with profilin significantly increased the PO activity (Figure 9C).

### Confocal Microscopy of NF-κB Distribution in Earthworm Seminal Vesicles

**Figure 10:**
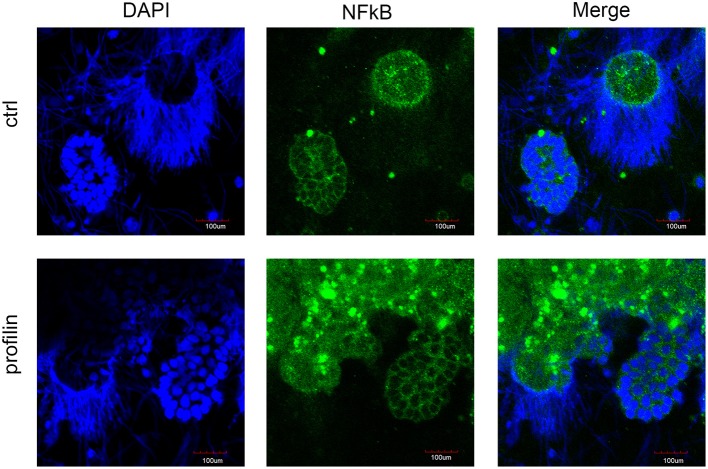
Confocal microscopy of NF-κB localization in seminal vesicles smears. Confocal microscopy showed the distribution of NF-κB p65 by immunofluorescence staining in control tissue and in tissue stimulated with profilin antigen. Blue corresponds to nuclear staining by DAPI and green corresponds to NF-κB p65 staining with antibody against NF-κB p65. Original magnification under ×40. After the stimulation with profilin, NF-κB was not translocated to the nucleus suggesting it is not involved in the signaling pathway via NF-κB. Scale bars represent 100 μm. Strongly stained spots correspond to the presence of melanin in cells.

In order to elucidate the potential involvement of mcc*Ea*TLR after the profilin stimulation of seminal vesicles in the canonical NF-κB pathway, we followed the distribution of NF-κB p65 in cells of control SV tissue and tissue treated with profilin antigens. NF-κB p65 was found to be distributed only in the cytoplasm of spermatic as well as supporting cells of SV. We did not detect any translocation of this factor to the nucleus after the antigenic treatment suggesting that profilin antigen did not activate NF-κB pathway in SV tissue (Figure 10).

### High-Throughput Sequencing of Gregarines Found in Earthworm Seminal Vesicles

The number of total sequence reads obtained by amplicon sequencing were approximately 28,582 ± 11,563 for the combination of primers SSU1f/SSU1r, and 18,587 ± 5,980 for the combination of primers SSU2f/Api (Table 3). Community evenness (>0.2), as well as the estimated diversity (Shannon-Wiener index), were similar in all samples from both sequence fragments (>0.5, Table 3).

**Table 3:** The alpha diversity parameters of samples from HTS.

	**SSU1f/SSU1r**	**SSU2r/Api1r**
Shannon-Wiener Diversity Index	0.502 ± 0.2	0.536 ± 0.184
Shannon entropy	0.724 ± 0.29	0.773 ± 0.266
Species richness (S)	10.58 ± 3.83	9.667 ± 2.605
Simpson diversity index	0.697 ± 0.15	0.651 ± 0.149
Evenness	0.215 ± 0.07	0.241 ± 0.084
Species Richness−80% diversity	1.5 ± 0.52	1.583 ± 0.515
Chao-1	17.93 ± 10.29	19.125 ± 4.991
Number of reads	*28, 582*±11, 563	*18, 587*±5, 980

*The diversity values were calculated from 6,000 subsampled sequences for both combinations of primers used for sequencing of gregarine 18S rDNA from seminal vesicles of E. andrei earthworms*.

The sequences of the gregarine 18S of both fragments clustered into 40 or 22 OTUs, respectively, at a 97% similarity threshold after excluding singletons. The longer fragment (SSU2f/Api, 477 bp) was used for the phylogenetic analysis.

**Figure 11:**
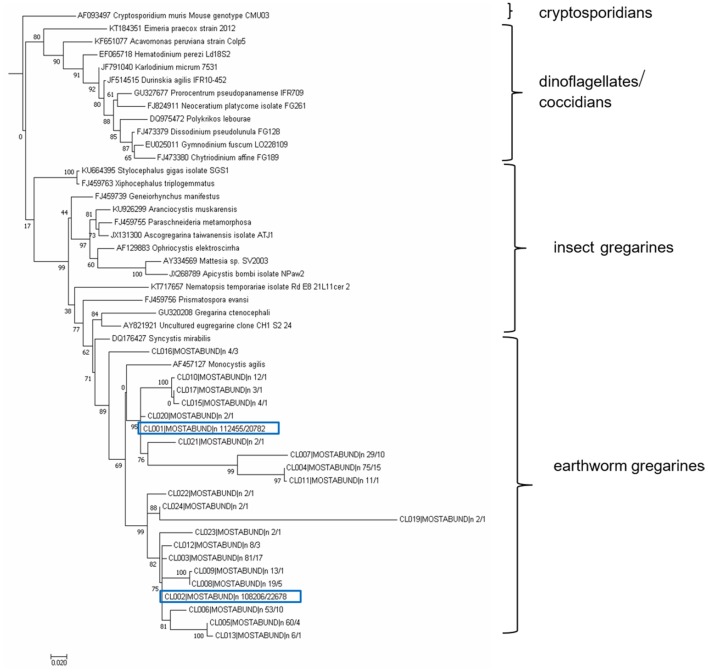
Maximum likelihood analysis of gregarine partial sequence of 18S rDNA. Obtained clustered sequences from a longer SSU region (SSU2/Api region, 477 bp) were compared with data from the NCBI GenBank using a BlastN similarity search. A matrix containing 49 SSU sequences were aligned in MAFFT 6 using the G-INS-i strategy (45). The maximum likelihood (ML) phylogenetic analyses were performed in PHYML (33) using default settings and 500 bootstrap replicates with the K2 + G model determined using MEGA 6.06 (34). The tree was rooted with *Cryptosporidium muris*. Earthworm gregarine sequences clustered with other earthworm gregarine species, suggesting their host specificity.

The resulting OTUs form a well-supported phylogenetic clade (bootstrap support, BS = 89) together with an annelid gregarine *Monocystis agilis*, a clade with a sister of another annelid gregarine *Syncystis mirabilis*, and with each forms a clade of gregarines specific to earthworms (Figure 11). Phylogenetically, they belong to the larger clade of insect gregarines (BS = 99). The recognized OTUs were further clustered into at least six main lineages, indicating greater diversity, probably covering several genera of organisms or strains of *Monocystis* sp. (Figure 11).

Depending on the SSU marker used, 7-19 OTUs, with a predicted total diversity reaching 37 OTUs (Chao-1 estimate) per single earthworm individual were found (Table S2 in Supplementary Material). The calculated alpha diversity, as well as rarefaction analysis of the OTUs, achieved in individual samples, are shown in Table S2 in Supplementary Material. A pairwise distance of all clusters ranges from 0.004 to 0.552 (Table S3 in Supplementary material).

## Discussion

The first TLR from Annelida were retrieved from *in silico* analyses of whole genomes of the polychaete *Capitella capitata* and the leech *Helobdella robusta* (49). From their genomes, 105 and 16 TLR homologs were found, respectively. The TLR receptors and their downstream signaling were well described in the medicinal leech *Hirudo medicinalis*, where TLR molecules are expressed in their central nervous system (CNS) (50). The first TLR isolated from *E. andrei* (*Ea*TLR) (3) has very large intra-species variability with a large number of TLR genes in the genome. In comparison to this *Ea*TLR, a newly described “multiple cysteine cluster” type TLR (mcc*Ea*TLR) has no variability and no introns. The comparison of amino acid sequences between both molecules revealed only 23% homology. Further, both receptors differ in their tissue expression. *Ea*TLR is expressed in all tissues of the earthworm body with the greatest constitutive expression in the digestive tract, and the mcc*Ea*TLR molecule is expressed primarily in earthworm seminal vesicles and seminal receptacles. Seminal vesicles are male reproductive organs where sperm cells develop. Seminal receptacles are female reproductive organs where sperm cells from the mating partner are stored. Since both organs have different origins, the expression of mcc*Ea*TLR is rather connected to the sperm cells. Importantly, earthworms are endowed with both multiple cysteine and single cysteine cluster TLRs according to the arrangement of their extracellular domain (Figure 1). Each earthworm TLR clusters with TLRs of other animals of corresponding types, and form two separate evolutionary branches (Figure 2).

Both receptors also differ in their abundance. Since *Ea*TLR is expressed at a common level, the mcc*Ea*TLR absolute numbers are very low (Figure 3B). The Toll of *Drosophila* was originally identified as a molecule playing a role in embryonal development (51). In the sea anemone *Nematostella vectensis*, knockdown of mcc type TLR led to the abnormal embryonic changes, suggesting its participation in early development (52). Further, molluscan TLRs likely contribute to the molluscan development, as was demonstrated by the upregulation of three *Crassostrea gigas* TLRs during embryonic development (53). The possible involvement of TOL-1 in the development is considered also in *Caenorhabditis elegans*, however, the downstream pathways activated by TOL-1 during early development is not elucidated yet (54).

Therefore, we tracked the mRNA levels of both receptors during earthworm development. *Ea*TLR was expressed mainly in older stages, from around 14 days, but mcc*Ea*TLR was expressed mainly in the early stages of earthworm life, suggesting its role in the early stages of earthworm development (Figures 3E,F). We are aware that the different proportions of reproductive organs to the whole body can influence the assessment of mcc*Ea*TLR mRNA levels during development. However, nearly all of the protostomian *Drosophila* Toll orthologs are involved in the regulation of embryogenesis; similarly, mcc*Ea*TLR can have primarily the developmental function. It is supported also by the fact that the expression of mcc*Ea*TLR is concentrated to the sites of gametogenesis.

The seminal vesicles of earthworms are generally deeply infected by gregarine parasites. Gregarines belong to the phylum Apicomplexa, which includes many protozoan parasites of medical and veterinary importance including *Plasmodium* (which causes malaria), *Toxoplasma*, and *Cryptosporidium*. Because gregarines do not parasitize vertebrates and do not cause serious damage to their hosts, many researchers do not explore them. During our studies, all tested individual earthworms exhibited seminal vesicles with gregarines, indicating heavy infection. Large oocysts were very often surrounded by cells with a strong melanization reaction, the prominent earthworm immune response (Figure 4). The melanization reaction is the consequence of a prophenoloxidase enzyme cascade. It is a part of the pathogen pacifying process common to many invertebrate animal groups, consisting of separation and encapsulation of the object within melanin-rich cell aggregates (24). Interestingly, the seminal vesicles are full of earthworm bristles enclosed with cells with a melanization reaction, resembling some gregarine development stages or nematode larvae, and can be easily mistaken (Figure S3 in Supplementary material). It is not clear how these bristles get into seminal vesicles, but they are recognized as non-self-objects activating prophenoloxidase cascade.

Previously, it was proposed that proPO cascade is initiated by earthworm PRR, coelomic cytolytic factor, CCF (55). CCF is expressed solely in gut tissue and coelomocytes, indicating the proPO cascade in seminal vesicles have to be activated through another mediator. Another PRR identified in earthworms, lipopolysaccharide-binding protein *Ea*LBI/BPI, is highly expressed in seminal vesicles and receptacles. Whether this molecule is involved in the activation of proPO cascade in seminal vesicles, further investigation would be needed. However, stimulation of seminal vesicles tissue with various antigens, but not of coelomocytes, led to an increase in *Ea*LBI/BPI mRNA levels (Figure 8D). Similarly, seminal vesicle lysate treated with profilin augmented PO activity suggesting a possible involvement of *Ea*LBI/BPI in earthworm defense mechanisms.

To prove the correlation between the presence of mcc*Ea*TLR on sperm cells and the occurrence of gregarines in seminal vesicles, earthworms were treated with two types of antibiotics, griseofulvin and metronidazole (Figure 5). In a previous study, infected grasshoppers with gregarine parasites living in their digestive tracts were fed lettuce containing these antibiotics (36). Earthworms cannot be fed in a similar way and, moreover, gregarines are found mainly in their seminal vesicles. So, large concentrations of antibiotics were put into the substrate where the earthworms were maintained, but the exact dose reaching the earthworm seminal vesicles could not be estimated. We assume that antibiotics entered the earthworm body through the dorsal pores and by the substrates consumed. Unfortunately, the antibiotics were administered in concentrations, which resulted in a significant decrease in the sperm cells of the earthworms. This is in agreement with findings that certain medications can have an adverse effect on sperm count and morphology (56). The destruction of sperm cells with the utilized antibiotics correlates with the decrease of the mcc*Ea*TLR mRNA levels, supporting the conclusion that mcc*Ea*TLR is expressed by sperm cells (Figure 7). We detected a decrease in the incidence of parasites, both at the level of sporocyst numbers and at the parasite DNA level after the antibiotic treatment. The damaging effect of the antibiotics on parasites is supported by the outcome of the antibiotic on sperm cells, because by their destruction gregarines lose their food. So, the decreased levels of mcc*Ea*TLR mRNA after the parasite treatment is a result of the decline of the sperm cell numbers rather than by diminution of parasites (Figures 5–7).

To obtain earthworms without parasites, we bred earthworms from cocoons, which were treated with antibiotics, in semi-sterile conditions without gregarines. To our surprise, gregarine DNA was found in these individuals in their seminal vesicles. This finding is inconsistent with the results of the study by Field and Michiels, who reported a successful method to obtain infection-free individuals (57). Nevertheless, they checked for the presence of parasites in seminal vesical smears by the use of a field microscope. For our analysis, we designed primers amplifying only gregarine DNA, which were subsequently utilized in qPCR. By this sensitive method, we detected gregarine DNA from samples containing very small amounts, which was the case for earthworms grown from cocoons. The specificity of the PCR products was confirmed by sequencing. Since parasite DNA was also found in cocoons but was absent in seminal receptacles, we concur with Field and Michiels that acephaline gregarine parasites (*Monocystis* sp.) are probably not transmitted sexually during earthworm mating (57). On the other hand, their occurrence in cocoons indicates another manner of infection. Copulation and reproduction take place separately in earthworms. The earthworm pair overlap front ends ventrally and they exchange their sperm. Sometime after their separation, they secrete a substance from the clitellum forming a ring around the worm. We propose that parasites clinging to the earthworm body get into cocoons when the worm backs out of the ring, which is then slipped out from the body. To follow the mRNA levels of receptors in cells after the microbial challenge, we stimulated worms with Gram-positive and Gram-negative bacteria. In the preceding study, we observed the upregulation of *Ea*TLR in coelomocytes after the administration of Gram-positive bacteria (3). The mRNA level of new mcc*Ea*TLR, which has a very low basal expression in coelomocytes, was not altered after the bacterial treatment (data not shown). Many TLRs are not transcriptionally regulated during the immune response, particularly in the case of long-term parasite infections. Alternatively, parasites can even downregulate TLRs expression (58, 59).On the other hand, *in vitro* stimulation of seminal vesicles with profilin, an antigen from the related parasite *T. gondii* proven as an antigen for mouse TLR11 and 12, led to the upregulation of mcc*Ea*TLR (Figure 8). This increase of mcc*Ea*TLR mRNA level was very short-term and it was not detectable already after 24 h (data not shown). Thus, the expression of mcc*Ea*TLR in seminal vesicles is not conditioned by the presence of parasites, but after the boost with parasite antigen, it can be augmented transiently. Such an increase was not detected after the profilin stimulation of coelomocytes. However, we have to admit the possibility that such an increase can reflect a secondary aspect of the immune response.

The Toll of *Drosophila*, the protostomian TLR type, requires proteolytic processing of the secreted polypeptide Spätzle for its activation. Its cleaved form then acts as a ligand for Toll (60). The coelomic fluid of earthworms contains a huge amount of proteolytic enzymes with strong proteolytic activity (61). Therefore, for our *in vitro* stimulation of SV tissue and cells, we supplemented cultivation medium with coelomic fluid. Since the augmentation of mcc*Ea*TLR mRNA level occurs only when coelomic fluid is added to the cultivation medium as a supplement, we suppose the involvement of other molecules, e.g., proteolytic enzymes, coming from the coelomic fluid in mcc*Ea*TLR regulation.

Although, components associated with TLR-to NF-kB signaling pathway were found in *E. andrei* earthworms (data not shown), including adaptor molecule MyD88, nuclear factor kappa B (NF-kB), and its inhibitor IkB, the mechanisms of the potential downstream signal transduction is not known. The presence of these signaling molecules implies that *Ea*TLRs can initiate the NF-kB-dependent signaling pathway, however, stimulation of seminal vesicles tissue with profilin did not lead to the translocation of NF-κB p65 to the nucleus, as was shown by confocal microscopy (Figure 10) and most probably it is not involved in the signaling pathway via NF-κB. Furthermore, *in vitro* antigen stimulation of neither seminal vesicles nor coelomocytes induced changes in mRNA levels of NF-κB molecules (Figure 8F).

Expression analysis of various defense and signaling molecules in seminal vesicles and coelomocytes after the antigenic stimulation revealed that all genes clustered according to their patterns. Both niches clustered separately indicating their different expression profiles (Figure 8H). Interestingly, the expression of endothelial monocyte-activation polypeptide II (EMAPII) correlated with the expression of mcc*Ea*TLR in seminal vesicles. It was described that TLRs regulate endothelial monocyte-activating polypeptide II (EMAPII) production upon microbial challenge in both mammals (13) and leech (14). However, the mRNA levels of *Ea*EMAP were not significantly increased following the antigenic stimulation (Figure 8C).

To estimate the amounts of parasites in the various tissues using a molecular approach, we designed specific primers amplifying only parasitic DNA and not the host DNA. Since the 18S rRNA sequences of both gregarine and the *E. andrei* earthworm are very similar, we could not employ primers used in previous studies in other earthworm species (62). The most common earthworm apicomplexan parasite is *Monocystis* sp., described mainly in *L. terrestris* species. The only examination of the diversity of *Monocystis* sp. at the genetic level was performed by cloning of PCR products and Sanger sequencing (62). Substantial diversity of the *Monocystis* genotypes was detected within a single host organism (62). Primers employed from this study, specific to the ribosomal ITS sequences, led to the amplification of *Eisenia* DNA. By HTS of 2 parasite 18S DNA segments, amplified with our primers specific to gregarine DNA, we obtained 40 or 22 OTUs at a 97% sequence similarity threshold. Phylogenetic analysis revealed that all sequences cluster with another earthworm gregarine *Monocystis agilis* and *Syncystis mirabilis*, suggesting their host specificity. Since there are few earthworm gregarine sequences available in the database, it is unfeasible to decide whether individual *E. andrei* earthworms were infected by a variety of *Monocystis agilis* strains or various *Monocystis* species, or several gregarine species.

## Conclusions

Earthworms do not possess adaptive immunity and they depend on innate immunity mechanisms represented by pattern recognition receptors (PRRs). Such types of receptors recognize conserved microbial molecular patterns and the most examined molecules are Toll-like receptors (TLRs). Here, we report that earthworms possess both types of TLRs distinguished according to the arrangement of their extracellular binding domains. Further, a newly described earthworm multiple cysteine cluster TLR is expressed by sperm cells and it is suggested to play a role in the early development of earthworms and potentially in the immune response against parasites. The seminal vesicles of earthworms are massively infected by gregarines, Apicomplexan protozoan parasites which feed on developing spermatocytes. With the exception of infections of seminal vesicles, which are contracted via the digestive tract by eating soil with spores, the parasites can be transferred into a new individual during the cocoon formation. Every individual earthworm has a diverse repertoire of parasitic genotypes in their seminal vesicles, corresponding to the variety of *Monocystis agilis* strains or various *Monocystis* species or several gregarine species. Parasites, as well as profilin antigen, activate prophenoloxidase cascade in earthworm seminal vesicles, the efficient mechanism of earthworm innate immunity. The expression of mccEaTLR in seminal vesicles correlates with the expression of inflammatory cytokine (EMAPII).

## Author Contributions

PP and MB conceived the project. PP, RR, and JD designed the study. FS contributed to TLR sequencing. JD performed histological staining. NP performed qPCR analysis. MK performed the phylogenetic analysis. MB contributed to the manuscript revision and supervised the study. PP prepared amplicon library for HTS. All authors read and approved the final manuscript.

### Conflict of Interest Statement

The authors declare that the research was conducted in the absence of any commercial or financial relationships that could be construed as a potential conflict of interest.

## Footnotes

^1^Elsworth B. *Earthworm Genome Sequencing Consortium. Blaxter, M., The Lumbricus rubellus Genome Project*. Available online at: www.earthworms.org.
