# Supplementary material for: Developmental and Immune Role of a Novel Multiple Cysteine Cluster TLR From Eisenia andrei Earthworms
Source: Front Immunol. 2019 Jun 18;10:1277. doi: 10.3389/fimmu.2019.01277 (PMC6591376; doi:10.3389/fimmu.2019.01277)
Supplement: Supplementary file 1 [file Data_Sheet_1.pdf]

Supplementary Material

1 Supplementary Figures

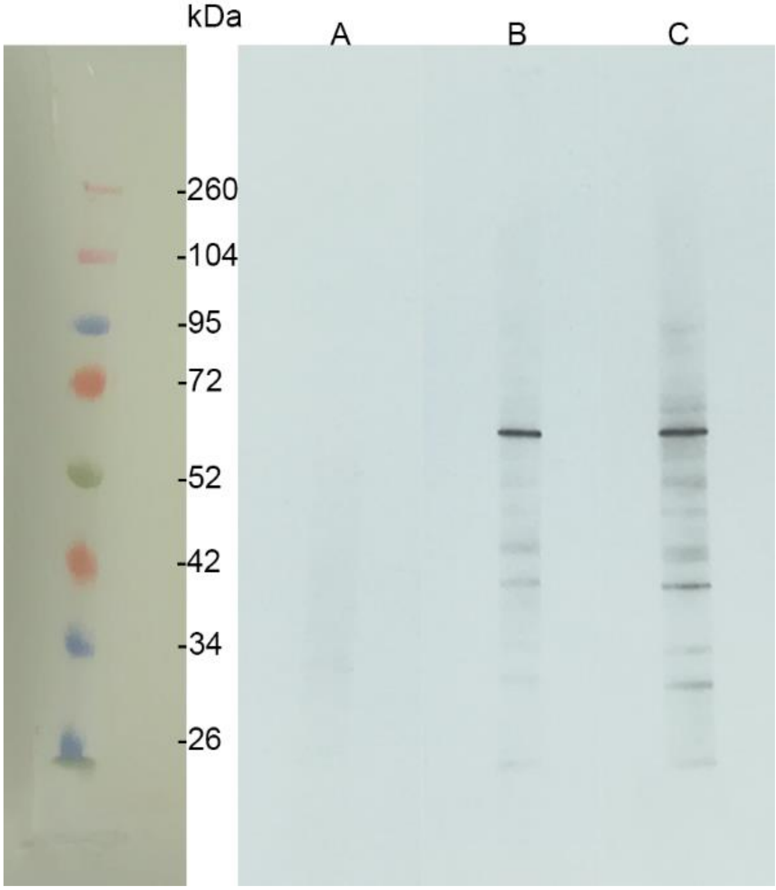

Supplementary Figure 1. Western blot analysis of seminal vesicles protein lysate probed with rabbit anti- NF-κB p65 antibody followed by detection with HRP conjugated goat anti-rabbit IgG. Seminal vesicles from 8 adult earthworms were dissected and cultivated in 60% RPMI medium with the antibiotic mixture for 6 hours with (ctrl) or without profilin antigen. Tissues were lysed in T-Per Tissue protein extraction reagent (Thermo Scientific) with proteinase inhibitor Arrest (Thermo Scientific) in combination with bead-beating (lysing matrix D, MPG). After 30 minutes incubation on ice, samples were centrifuged for 20 minutes (14000 rpm) and supernatants were separated in 12% SDS PAGE under reducing conditions. After separation, the proteins were electroblotted to nitrocellulose membrane (Hybond-C pure, Amersham). The membranes were blocked with 2% low-fat milk in PBS-T for 1h at RT and then incubated with rabbit anti- NF-κB p65 antibody (D14E12, Cell Signaling; 1:1000 diluted in blocking solution) overnight at 4 °C. After washing with PBS-T, HRP conjugated goat anti-rabbit IgG (7074, Cell Signaling; 1:10000 diluted in blocking solution) was applied to the membranes for 1h at RT. Chemiluminescence reagents (SuperSignal West Pico kit, Thermo Scientific) and X-ray film (Carestream) were used for visualization of the binding of Ab specific for the NF-κB. A) no primary antibody added, B) protein lysate from non-treated seminal vesicles, C) protein lysate from seminal vesicles treated with profilin antigen.

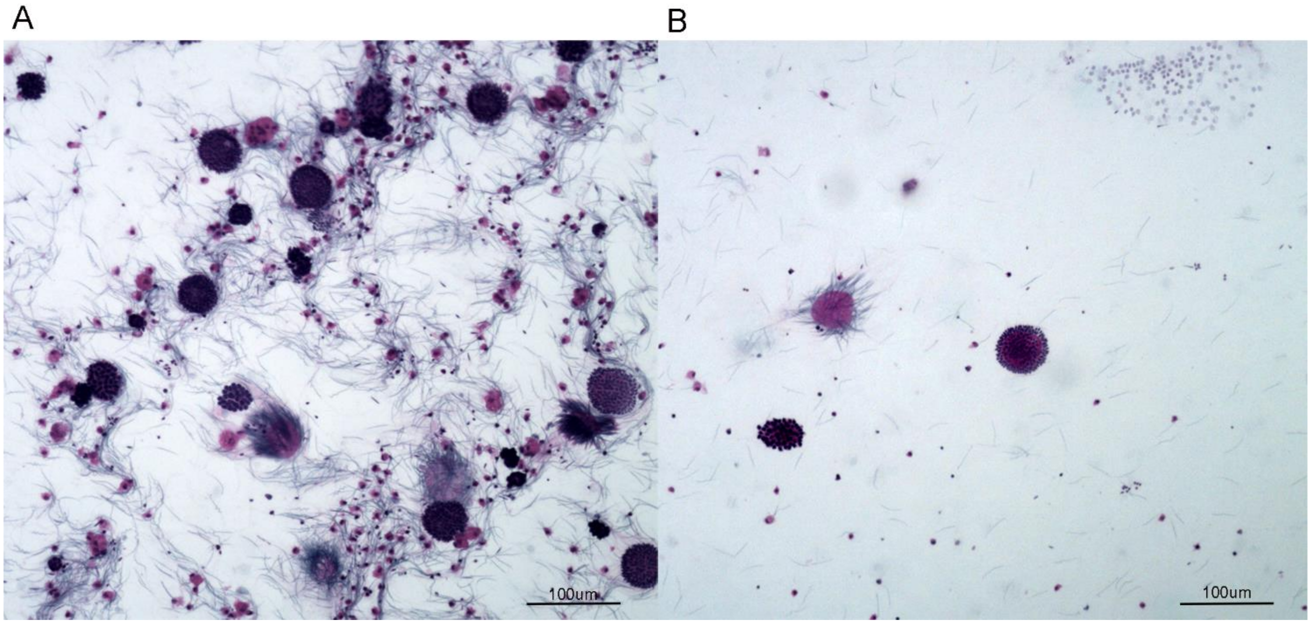

Supplementary Figure 2. Smears of earthworm seminal vesicles**. (**A) a typical smear of seminal vesicles before antibiotic treatment, (B) reduced the number of sperm cells as well as all forms of developing spermatocytes as a consequence of antibiotic treatment. Stained by Hematoxylin/Eosin.

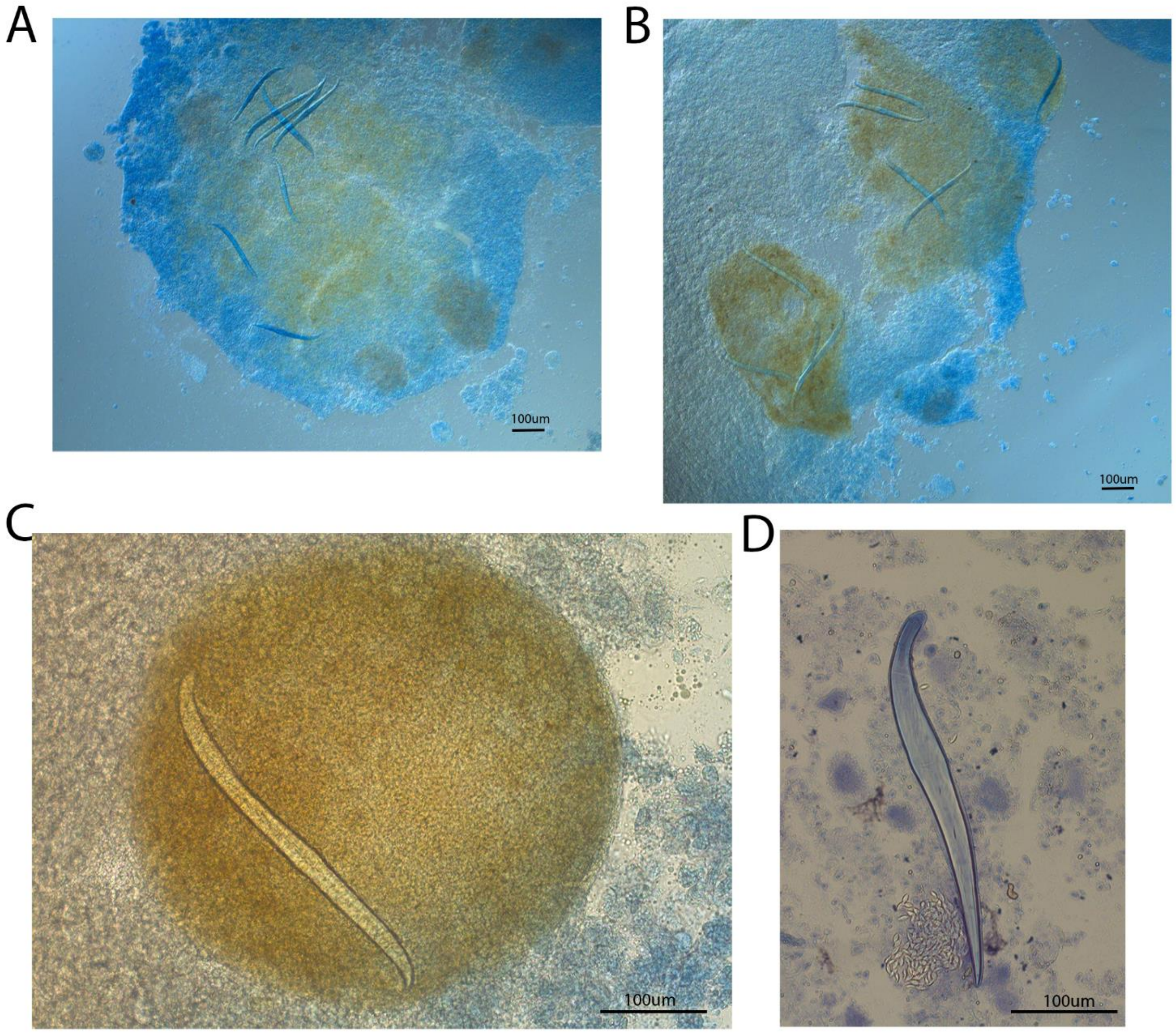

Supplementary Figure 3. Earthworm bristles in seminal vesicles. (A, B, C) bristles in seminal vesicle tissues surrounded by melanization reaction, stained with Methylene Blue, (D) bristle associated with released sporocysts, stained with Trypan Blue.

Supplementary Tables

Supplementary Table S1. Primers for gregarine 18S rRNA used for HTS.

Primers for gregarine 18S rRNA		
Name	Direction	Sequence
SSU1f	forward	5′-XXXXXX*CC*GCACCATGCATGTCTAAGTATAAGTT-3′
SSU2f	forward	5′-XXXXXX*AC*AGTTGTCAATCAAATGACTCTTTC-3′
SSU1r	reverse	5′-XXXXXX*GC*TGCAAGCATAGGTTGGTTCT-3′
Api1r	reverse	5′-XXXXXX*CC*TAATCTATCCCCATCACGATGC-3′
Combinations of primers		Size of amplicons (bp)
341F/806R		465
SSU1f/SSU1r		200
SSU2f/Api1r		477

X represents a barcode base, linkers are in *italic*

Supplementary Table S2.

**Combination of primers: SSU1f/SSU1r**												
	**SV_01**	**SV_02**	**SV_03**	**SV_04**	**SV_05**	**SV_06**	**SV_07**	**SV_08**	**SV_09**	**SV_10**	**SV_11**	**SV_12**
Shannon-Wiener Diversity Index	0.47	0.22	0.73	0.69	0.34	0.46	0.69	0.72	0.18	0.56	0.29	0.67
Shannon Entropy	0.67	0.32	1.06	0.99	0.49	0.66	0.10	1.04	0.26	0.80	0.42	0.97
Species Richness (S)	9	12	12	10	7	13	19	13	4	9	7	11
Total Abundance	6000	6000	6000	6000	6000	6000	6000	6000	6000	6000	6000	6000
Simpson Diversity Index	0.73	0.90	0.50	0.54	0.83	0.75	0.58	0.54	0.92	0.67	0.86	0.56
Evenness	0.21	0.09	0.29	0.30	0.18	0.18	0.23	0.28	0.13	0.25	0.15	0.28
Species Richness - 80% diversity	1	1	2	2	1	1	2	2	1	2	1	2
Chao-1	14	33	27	10.33	8	20.5	37.33	18	4	12	10	21
Number of reads	34381	23201	31426	22049	30157	8942	51871	30116	42017	11324	49491	8013

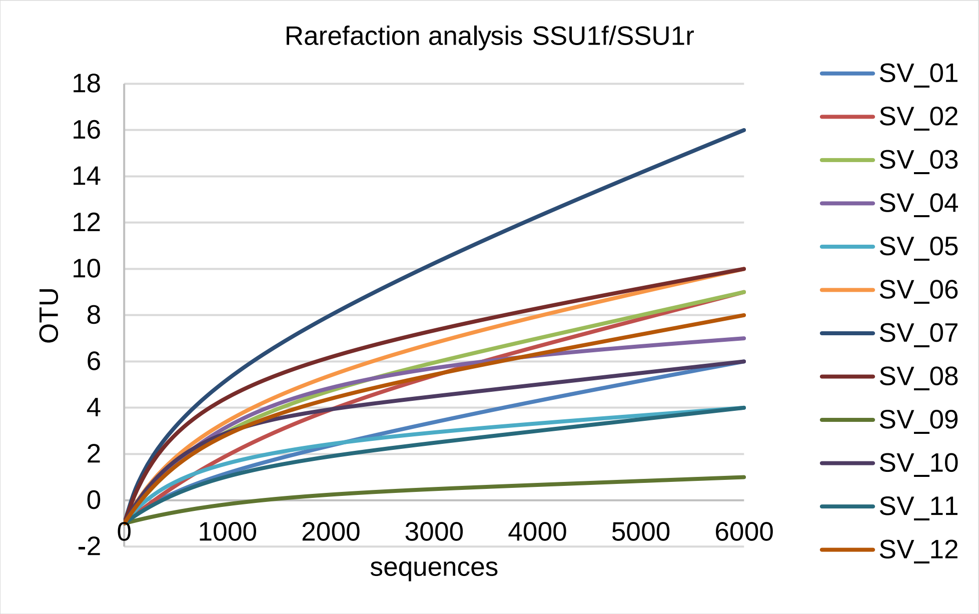

**Combination of primers: SSU2f/Api1r**												
	**SV_01**	**SV_02**	**SV_03**	**SV_04**	**SV_05**	**SV_06**	**SV_07**	**SV_08**	**SV_09**	**SV_10**	**SV_11**	**SV_12**
Shannon-Wiener Diversity Index	0.70	0.50	0.52	0.41	0.67	0.67	0.26	0.15	0.51	0.65	0.71	0.68
Shannon Entropy	1.01	0.72	0.76	0.60	0.95	0.97	0.37	0.21	0.74	0.94	1.02	0.98
Species Richness (S)	8	7	9	7	13	9	15	7	11	12	10	8
Total Abundance	6000	6000	6000	6000	6000	6000	6000	6000	6000	6000	6000	6000
Simpson Diversity Index	0.50	0.69	0.67	0.76	0.55	0.53	0.88	0.94	0.69	0.56	0.51	0.52
Evenness	0.34	0.26	0.24	0.21	0.26	0.31	0.10	0.07	0.21	0.26	0.31	0.33
Species Richness - 80% diversity	2	1	2	1	2	2	1	1	1	2	2	2
Chao-1	18	13	19	17	31	19	27	17	18	17	15	18
reads	33165	27794	23506	23154	20053	19407	18919	17851	11711	11486	9506	6502

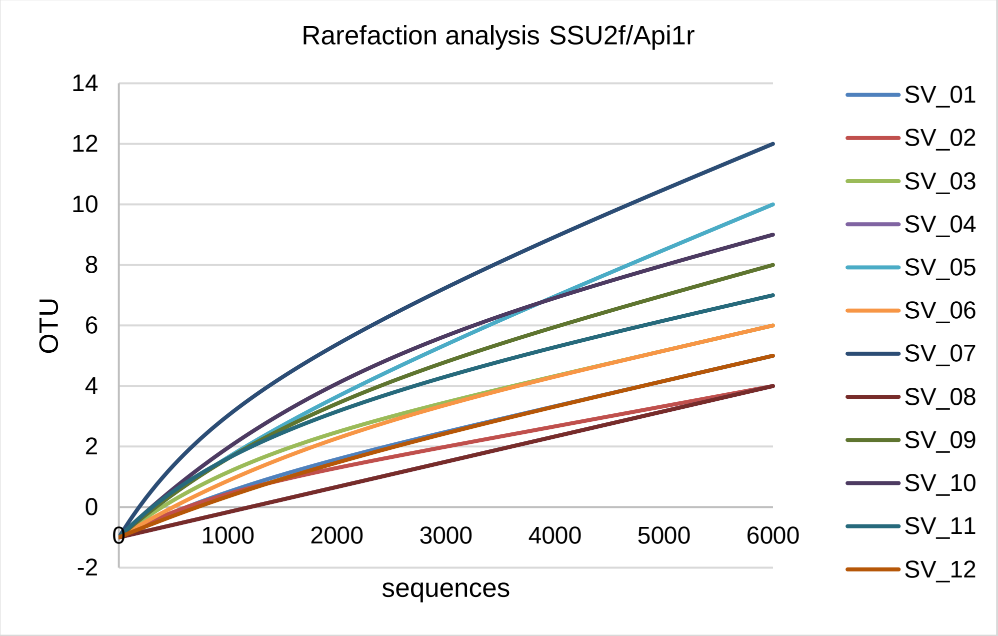

Supplementary Table S2. Alpha diversity and rarefaction analysis of samples from HTS. The alpha diversity and rarefaction analysis of OTUs based on gregarine 18S rRNA from seminal vesicles of *E. andrei* earthworms were counted from 6000 subsampled sequences for both combinations of primers. OTUs were generated at 97% identity.

Supplementary Table S3. Estimates of evolutionary divergence between sequences obtained from SSU2/Api fragments.

**A**																					
CL001|n_112455/20782																					
CL020|n_2/1	0.0046																				
CL010|n_12/1	0.0283	0.0352																			
CL017|n_3/1	0.0283	0.0352	0.0047																		
CL015|n_4/1	0.0482	0.0625	0.0080	0.0040																	
CL022|n_2/1	0.0348	0.0276	0.0660	0.0660	0.1124																
CL021|n_2/1	0.0306	0.0394	0.0595	0.0619	0.1061	0.0635															
CL002|n_108206/22678	0.0499	0.0435	0.0707	0.0707	0.0963	0.0326	0.0829														
CL012|n_8/3	0.0559	0.0498	0.0710	0.0710	0.0971	0.0381	0.0900	0.0089													
CL003|n_81/17	0.0514	0.0473	0.0731	0.0731	0.0978	0.0381	0.0833	0.0044	0.0138												
CL009|n_13/1	0.0696	0.0643	0.0731	0.0731	0.1004	0.0501	0.1028	0.0281	0.0312	0.0313											
CL024|n_2/1	0.0512	0.0410	0.0546	0.0511	0.0580	0.0238	0.1034	0.0102	0.0102	0.0178	0.0136										
CL008|n_19/5	0.1004	0.0933	0.0590	0.0545	0.0593	0.0645	0.1626	0.048	0.0413	0.0550	0.004	0,00									
CL006|n_53/10	0.0717	0.0666	0.0945	0.0945	0.1370	0.0392	0.1028	0.0216	0.0313	0.0268	0.05	0.0443	0.0883								
CL023|n_2/1	0.0760	0.0712	0.0943	0.0943	0.0963	0.0586	0.1094	0.0325	0.0425	0.0380	0.0586	0.0238	0.0481	0.0543							
CL016|n_4/3	0.0607	0.0619	0.0754	0.0754	0.1004	0.0739	0.0938	0.0649	0.0691	0.0669	0.0911	0.0714	0.12	0.0867	0.0889						
CL005|n_60/4	0.0881	0.0839	0.1116	0.1142	0.1780	0.0604	0.0928	0.0348	0.0455	0.0407	0.0651	0.0684	0.1232	0.0414	0.0697	0.1020					
CL013|n_6/1	0.1010	0.1014	0.1333	0.1375	0.4	0.1014	0.1010	0.0613	0.0798	0.0649	0.1050	0.1651	0.4153	0.0615	0.1159	0.1083	0.0072				
CL007|n_29/10	0.0991	0.0924	0.1441	0.1472	0.3178	0.1022	0.1019	0.1377	0.1489	0.1461	0.1629	0.2153	0.3443	0.1325	0.1712	0.1432	0.1114	0.0929			
CL004|n_75/15	0.1176	0.1066	0.1784	0.1784	0.5106	0.1180	0.1158	0.1535	0.1678	0.1568	0.1803	0.2826	0.5212	0.1540	0.1934	0.1633	0.0871	0.0830	0.0774		
CL011|n_11/1	0.1111	0.0985	0.1606	0.1606	0.3769	0.1143	0.1124	0.1461	0.1554	0.1554	0.1730	0.2356	0.3923	0.1466	0.1818	0.1491	0.0866	0.0866	0.0721	0.0032	
CL019|n_2/1	0.1730	0.1735	0.1736	0.1701	0.0669	0.1453	0.2272	0.1344	0.1344	0.1485	0.1379	0.056	0.0090	0.1695	0.1379	0.1931	0.2084	0.5238	0.4031	0.5522	0.447058823529412

B

CL001|MOSTABUND|n_112455/20782																																																
CL020|MOSTABUND|n_2/1	0.0045																																															
CL010|MOSTABUND|n_12/1	0.0283	0.0351																																													
CL017|MOSTABUND|n_3/1	0.0283	0.0351	0.0047																																												
CL015|MOSTABUND|n_4/1	0.0481	0.0625	0.0080	0.0040																																										
DQ176427_Syncystis_mirabilis	0.0563	0.0574	0.0566	0.0566	0.0722																																									
AY821921_Uncultured_eugregarine_clone_CH1_S2_	0.0697	0.0715	0.0781	0.0781	0.0722	0.0370																																								
AF457127_Monocystis_agilis	0.0520	0.0574	0.0707	0.0707	0.0843	0.0780	0.0871																																							
CL022|MOSTABUND|n_2/1	0.0347	0.0276	0.0660	0.0660	0.1124	0.0782	0.0917	0.0782																																						
CL021|MOSTABUND|n_2/1	0.0306	0.0393	0.0595	0.0619	0.1061	0.0875	0.1010	0.0787	0.0635																																					
CL002|MOSTABUND|n_108206/22678	0.0498	0.0435	0.0707	0.0707	0.0963	0.0672	0.0697	0.0694	0.0326	0.0829																																				
CL012|MOSTABUND|n_8/3	0.0559	0.0497	0.0710	0.0710	0.0971	0.0648	0.0696	0.0782	0.0381	0.0900	0.0089																																		
CL003|MOSTABUND|n_81/17	0.0514	0.0473	0.0731	0.0731	0.0978	0.0693	0.0719	0.0715	0.0381	0.0833	0.0044	0.0138																																
CL024|MOSTABUND|n_2/1	0.0511	0.0410	0.0546	0.0511	0.0580	0.0580	0.0648	0.0648	0.0238	0.1034	0.0102	0.0102	0.0178																															
CL009|MOSTABUND|n_13/1	0.0695	0.0643	0.0731	0.0731	0.1004	0.0913	0.0960	0.0891	0.0501	0.1028	0.0281	0.0312	0.0313	0.0136																														
CL008|MOSTABUND|n_19/5	0.1004	0.0933	0.0590	0.0545	0.0593	0.1044	0.1004	0.1164	0.0645	0.1626	0.048	0.0413	0.0550	0,000	0.004																													
CL006|MOSTABUND|n_53/10	0.0717	0.0666	0.0945	0.0945	0.1370	0.0891	0.0917	0.0913	0.0392	0.1028	0.0216	0.0313	0.0268	0.0443	0.05	0.0883																												
CL023|MOSTABUND|n_2/1	0.0760	0.0712	0.0943	0.0943	0.0963	0.0913	0.0982	0.0934	0.0586	0.1094	0.0325	0.0425	0.0380	0.0238	0.0586	0.0481	0.0543																											
CL016|MOSTABUND|n_4/3	0.0607	0.0619	0.0754	0.0754	0.1004	0.0607	0.0697	0.0845	0.0739	0.0938	0.0649	0.0691	0.0669	0.0714	0.0911	0.12	0.0867	0.0889																										
FJ459756_Prismatospora_evansi	0.0847	0.0898	0.0921	0.0921	0.0923	0.0695	0.0762	0.1021	0.1067	0.1140	0.0891	0.0874	0.0919	0.0716	0.1111	0.1124	0.1067	0.1132	0.1021																									
FJ459739_Geneiorhynchus_manifestus	0.0802	0.0827	0.0849	0.0849	0.0963	0.0498	0.0588	0.0954	0.1021	0.1050	0.0911	0.0850	0.0939	0.0784	0.1152	0.1244	0.1086	0.1152	0.0845	0.0695																								
GU320208_Gregarina_ctenocephali	0.0891	0.0944	0.0849	0.0801	0.0843	0.0695	0.0633	0.1	0.1176	0.1184	0.0956	0.0941	0.0986	0.0716	0.1176	0.125	0.1176	0.1176	0.1	0.0936	0.0847																							
FJ459755_Paraschneideria_metamorphosa	0.0780	0.0804	0.0778	0.0778	0.0883	0.0585	0.0697	0.0889	0.1	0.1072	0.0911	0.0805	0.0939	0.0716	0.1086	0.1244	0.1086	0.1152	0.0889	0.0847	0.0498	0.0826																						
JX131300_Ascogregarina_taiwanensis_isolate_ATJ1	0.0802	0.0827	0.0872	0.0872	0.0883	0.0585	0.0718	0.0932	0.1021	0.1094	0.0932	0.0894	0.0961	0.0750	0.1108	0.1164	0.1108	0.1152	0.0867	0.0739	0.0520	0.0891	0.0216																					
KU926299_Aranciocystis_muskarensis	0.0824	0.0850	0.0896	0.0896	0.1004	0.0672	0.0762	0.0954	0.1	0.1115	0.0932	0.0894	0.0939	0.0784	0.1108	0.1204	0.1108	0.1173	0.0889	0.0891	0.0520	0.0934	0.0281	0.0260																				
AY334569_Mattesia_sp._SV2003	0.0978	0.0990	0.1087	0.1063	0.0967	0.0847	0.0851	0.1108	0.1176	0.1271	0.1065	0.1165	0.1098	0.1058	0.1263	0.1285	0.1241	0.1285	0.1108	0.0958	0.0760	0.1089	0.0760	0.0739	0.0760																			
JX268789_Apicystis_bombi_isolate_NPaw2	0.1065	0.1082	0.1134	0.1111	0.0967	0.1021	0.1004	0.1239	0.1220	0.1359	0.1108	0.1165	0.1143	0.0955	0.1285	0.1204	0.1285	0.1328	0.1239	0.1002	0.0956	0.1154	0.0804	0.0826	0.0804	0.0347																		
AF129883_Ophriocystis_elektroscirrha	0.0932	0.0965	0.1014	0.1014	0.1004	0.0650	0.0806	0.0997	0.1152	0.1225	0.1019	0.1006	0.1051	0.0819	0.1239	0.1244	0.1195	0.1217	0.0976	0.0782	0.0563	0.0934	0.0412	0.0325	0.0412	0.0695	0.0673																	
KT717657_Nematopsis_temporariae_isolate_Rd_E8_	0.0980	0.0993	0.1066	0.1018	0.1129	0.0893	0.0895	0.1154	0.1157	0.1274	0.1089	0.1011	0.1123	0.0955	0.1331	0.1325	0.1266	0.1288	0.1045	0.1045	0.0784	0.1004	0.0915	0.0936	0.0915	0.1045	0.1067	0.0958																
CL005|MOSTABUND|n_60/4	0.0881	0.0839	0.1116	0.1142	0.1780	0.1020	0.1048	0.1020	0.0604	0.0928	0.0348	0.0455	0.0407	0.0684	0.0651	0.1232	0.0414	0.0697	0.1020	0.1162	0.1160	0.1302	0.1160	0.1183	0.1183	0.1302	0.1372	0.1276	0.1282																			
CL013|MOSTABUND|n_6/1	0.1010	0.1014	0.1333	0.1375	0.4	0.1299	0.1345	0.1227	0.1014	0.1010	0.0613	0.0798	0.0649	0.1651	0.1050	0.4153	0.0615	0.1159	0.1083	0.1557	0.1516	0.1630	0.1516	0.1552	0.1516	0.1702	0.1847	0.1660	0.1745	0.0072																		
CL007|MOSTABUND|n_29/10	0.0991	0.0924	0.1441	0.1472	0.3178	0.1570	0.1745	0.1460	0.1022	0.1019	0.1377	0.1489	0.1461	0.2153	0.1629	0.3443	0.1325	0.1712	0.1432	0.1850	0.1707	0.1961	0.1707	0.1735	0.1735	0.1906	0.2016	0.1900	0.1772	0.1114	0.0929																	
CL004|MOSTABUND|n_75/15	0.1176	0.1066	0.1784	0.1784	0.5106	0.1797	0.2006	0.1699	0.1180	0.1158	0.1535	0.1678	0.1568	0.2826	0.1803	0.5212	0.1540	0.1934	0.1633	0.2098	0.1960	0.2262	0.1960	0.1993	0.1928	0.2098	0.2295	0.2156	0.2203	0.0871	0.0830	0.0774																
CL011|MOSTABUND|n_11/1	0.1111	0.0985	0.1606	0.1606	0.3769	0.1608	0.1794	0.1578	0.1143	0.1124	0.1461	0.1554	0.1554	0.2356	0.1730	0.3923	0.1466	0.1818	0.1491	0.1935	0.1754	0.2023	0.1812	0.1842	0.1842	0.1964	0.2140	0.1988	0.1970	0.0866	0.0866	0.0721	0.0032															
KU664395_Stylocephalus_gigas_isolate_SGS1	0.1062	0.1080	0.1084	0.1037	0.0963	0.0911	0.0980	0.1193	0.1217	0.1356	0.1149	0.1096	0.1185	0.0887	0.1304	0.1204	0.1326	0.1326	0.1171	0.1130	0.0802	0.1	0.0824	0.0824	0.0889	0.0978	0.0978	0.0889	0.0915	0.1438	0.1949	0.1983	0.2352	0.2105														
FJ459763_Xiphocephalus_triplogemmatus	0.1062	0.1080	0.1084	0.1037	0.0923	0.0954	0.0980	0.1149	0.1217	0.1356	0.1149	0.1096	0.1185	0.0887	0.1304	0.1204	0.1326	0.1326	0.1171	0.1130	0.0802	0.0976	0.0780	0.0824	0.0845	0.0934	0.0934	0.0867	0.0893	0.1438	0.1949	0.1983	0.2352	0.2105	0.0043													
AF093497_Cryptosporidium_muris_Mouse_genotype	0.1106	0.1126	0.1179	0.1132	0.0883	0.1019	0.1089	0.1279	0.1282	0.1400	0.1106	0.1118	0.1140	0.0887	0.1347	0.1204	0.1282	0.1347	0.1171	0.1065	0.0911	0.1214	0.0889	0.0932	0.0932	0.1	0.0934	0.0911	0.1132	0.1392	0.1877	0.2093	0.2418	0.2192	0.0844	0.0842												
KT184351_Eimeria_praecox_strain_2012	0.1149	0.1149	0.1226	0.1179	0.1124	0.1062	0.1198	0.1301	0.1369	0.1444	0.1323	0.1230	0.1364	0.1194	0.1543	0.1526	0.15	0.1521	0.1279	0.1282	0.1062	0.1282	0.1106	0.1127	0.1127	0.1173	0.1282	0.1171	0.1002	0.1600	0.2057	0.2038	0.2352	0.2105	0.0952	0.0909	0.0995											
KF651077_Acavomonas_peruviana_strain_Colp5	0.0978	0.1013	0.1108	0.1061	0.1044	0.0934	0.1113	0.1239	0.1198	0.1271	0.1130	0.1121	0.1165	0.1058	0.1285	0.125	0.1307	0.1350	0.1065	0.1154	0.0891	0.1217	0.1	0.1043	0.1	0.1067	0.1045	0.1043	0.1157	0.1418	0.1811	0.1878	0.2196	0.1964	0.0760	0.0824	0.0865	0.0891										
DQ975472_Polykrikos_lebourae	0.1084	0.1103	0.1037	0.1037	0.1004	0.0932	0.1111	0.1149	0.1260	0.1400	0.1171	0.1073	0.1208	0.0819	0.1304	0.1164	0.1347	0.1369	0.1149	0.1108	0.0911	0.1108	0.0954	0.1019	0.0976	0.1152	0.1108	0.0976	0.1111	0.1508	0.1985	0.2066	0.2418	0.2163	0.0909	0.0865	0.1099	0.0974	0.0670									
EU025011_Gymnodinium_fuscum_LO228109	0.1062	0.1080	0.1108	0.1061	0.0963	0.0889	0.1089	0.1171	0.1282	0.1356	0.1214	0.1140	0.1252	0.0887	0.1369	0.1244	0.1391	0.1391	0.1127	0.1043	0.0867	0.1108	0.0845	0.0824	0.0780	0.0956	0.0891	0.0824	0.1089	0.1508	0.1985	0.2038	0.2320	0.2076	0.0909	0.0865	0.0991	0.0887	0.0606	0.0430								
FJ473379_Dissodinium_pseudolunula_FG128	0.1084	0.1103	0.1108	0.1061	0.1044	0.0867	0.1067	0.1127	0.1304	0.1378	0.1193	0.1118	0.1230	0.0921	0.1369	0.1285	0.1369	0.1391	0.1106	0.1021	0.0845	0.1130	0.0824	0.0802	0.0759	0.0934	0.0869	0.0780	0.1067	0.1484	0.1913	0.2066	0.2320	0.2076	0.0865	0.0822	0.0926	0.0865	0.0562	0.0387	0.0129							
GU327677_Prorocentrum_pseudopanamense_IFR70	0.1171	0.1195	0.1132	0.1132	0.1124	0.0911	0.1089	0.1236	0.1391	0.1466	0.1279	0.1208	0.1319	0.1023	0.1434	0.1405	0.1456	0.15	0.1193	0.1152	0.0954	0.1152	0.0911	0.0932	0.0911	0.1065	0.1021	0.0954	0.1089	0.1577	0.2057	0.2093	0.2418	0.2163	0.0930	0.0887	0.1077	0.0974	0.0649	0.0451	0.0408	0.0322						
JF514515_Durinskia_agilis_IFR10-452	0.1171	0.1172	0.1179	0.1179	0.1164	0.0932	0.1111	0.1193	0.1347	0.1466	0.1214	0.1163	0.1252	0.0955	0.1434	0.1365	0.1391	0.1413	0.1171	0.1086	0.0911	0.1108	0.0932	0.0911	0.0867	0.0934	0.0913	0.0889	0.1067	0.1508	0.1985	0.2121	0.2418	0.2163	0.0822	0.0779	0.0948	0.0844	0.0606	0.0408	0.0387	0.0258	0.0279					
JF791040_Karlodinium_micrum_7531	0.1193	0.1195	0.1202	0.1155	0.1164	0.0976	0.1132	0.1214	0.1369	0.1487	0.1258	0.1208	0.1297	0.0955	0.1456	0.1365	0.1434	0.1456	0.1214	0.1108	0.0954	0.1086	0.0954	0.0932	0.0889	0.0978	0.0934	0.0911	0.1045	0.1554	0.2057	0.2148	0.2450	0.2192	0.0779	0.0735	0.0928	0.0822	0.0584	0.0409	0.0366	0.0237	0.0280	0.0064				
FJ473380_Chytriodinium_affine_FG189	0.1106	0.1126	0.1108	0.1061	0.1044	0.0954	0.1154	0.1236	0.1326	0.1400	0.1258	0.1163	0.1297	0.0955	0.1347	0.1285	0.1434	0.1456	0.1193	0.1130	0.0932	0.1239	0.0867	0.0845	0.0802	0.1021	0.0956	0.0867	0.1198	0.1554	0.2021	0.2066	0.2352	0.2134	0.0932	0.0889	0.0930	0.0845	0.0541	0.0453	0.0259	0.0215	0.0453	0.0410	0.0388			
EF065718_Hematodinium_perezi_Ld18S2	0.1041	0.1080	0.1155	0.1108	0.1044	0.0976	0.1132	0.1171	0.1260	0.1334	0.1171	0.1208	0.1208	0.0955	0.1369	0.1285	0.1347	0.1347	0.1149	0.1130	0.0954	0.1065	0.1019	0.0976	0.0932	0.0978	0.0956	0.0889	0.1111	0.1461	0.1913	0.1983	0.2320	0.2076	0.0844	0.0800	0.0840	0.0909	0.0497	0.0516	0.0494	0.0473	0.0494	0.0344	0.0280	0.0475		
FJ824911_Neoceratium_platycorne_isolate_FG261	0.1149	0.1172	0.1132	0.1132	0.1084	0.1019	0.1198	0.1301	0.1369	0.1444	0.1323	0.1230	0.1364	0.1023	0.1478	0.1445	0.15	0.1543	0.1258	0.1239	0.1019	0.1173	0.0954	0.0954	0.0932	0.1043	0.1043	0.0997	0.1176	0.1600	0.2093	0.2093	0.2385	0.2163	0.1082	0.1038	0.1142	0.1060	0.0757	0.0473	0.0473	0.0494	0.0387	0.0365	0.0409	0.0496	0.0516	
CL019|MOSTABUND|n_2/1	0.1730	0.1735	0.1736	0.1701	0.0669	0.1764	0.1695	0.1868	0.1453	0.2272	0.1344	0.1344	0.1485	0.056	0.1379	0.0090	0.1695	0.1379	0.1931	0.1833	0.1972	0.1937	0.1937	0.1903	0.1972	0.2006	0.1903	0.1937	0.1903	0.2084	0.5238	0.4031	0.5522	0.4470	0.1903	0.1903	0.1903	0.2110	0.2041	0.1868	0.1972	0.1972	0.2041	0.1972	0.1972	0.2006	0.2027	0.2076

Supplementary Table S3. The number of base differences per site between sequences from found clusters and clusters with representative sequences of other species are shown. The analysis involved 22 (A) or 49 (B) nucleotide sequences. All ambiguous positions were removed for each sequence pair. There were 543 positions in the final dataset. Evolutionary analyses were conducted in MEGA7.
